# Comprehensive characterization of β-alanine metabolism-related genes in HCC identified a novel prognostic signature related to clinical outcomes

**DOI:** 10.18632/aging.205744

**Published:** 2024-04-16

**Authors:** Yi Jia, Xu Chen, Hui Guo, Biao Zhang, Bin Liu

**Affiliations:** 1 Department of General Surgery, Xinhua Hospital of Dalian University, Dalian, Liaoning, China; 2 Department of General Surgery, First Affiliated Hospital of Dalian Medical University, Dalian, Liaoning, China

**Keywords:** hepatocellular carcinoma, β-alanine metabolism, prognosis model, bulk RNA sequencing, single-cell RNA sequencing

## Abstract

Hepatocellular carcinoma (HCC) stands out as the most prevalent type of liver cancer and a significant contributor to cancer-related fatalities globally. Metabolic reprogramming, particularly in glucose, lipid, and amino acid metabolism, plays a crucial role in HCC progression. However, the functions of β-alanine metabolism-related genes (βAMRGs) in HCC remain understudied. Therefore, a comprehensive evaluation of βAMRGs is required, specifically in HCC. Initially, we explored the pan-cancer landscape of βAMRGs, integrating expression profiles, prognostic values, mutations, and methylation levels. Subsequently, scRNA sequencing results indicated that hepatocytes had the highest scores of β-alanine metabolism. In the process of hepatocyte carcinogenesis, metabolic pathways were further activated. Using βAMRGs scores and expression profiles, we classified HCC patients into three subtypes and examined their prognosis and immune microenvironments. Cluster 3, characterized by the highest βAMRGs scores, displayed the best prognosis, reinforcing β-alanine’s significant contribution to HCC pathophysiology. Notably, immune microenvironment, metabolism, and cell death modes significantly varied among the β-alanine subtypes. We developed and validated a novel prognostic panel based on βAMRGs and constructed a nomogram incorporating risk degree and clinicopathological characteristics. Among the model genes, EHHADH has been identified as a protective protein in HCC. Its expression was notably downregulated in tumors and exhibited a close correlation with factors such as tumor staging, grading, and prognosis. Immunohistochemical experiments, conducted using HCC tissue microarrays, substantiated the validation of its expression levels. In conclusion, this study uncovers β-alanine’s significant role in HCC for the first time, suggesting new research targets and directions for diagnosis and treatment.

## INTRODUCTION

In 2020, liver cancer ranked as the sixth most frequently identified cancer and stood as the third primary contributor to global cancer-related fatalities [1]. The survival rate for HCC patients is significantly compromised due to late disease presentation, which limits the possibility of curative surgical intervention for the majority of patients at intermediate or advanced stages. Chemotherapy plays a crucial role in the treatment of advanced HCC; however, the effectiveness of this therapeutic approach is often hampered by the development of drug resistance in a considerable number of patients [2, 3]. Despite advancements in therapeutic management, the prognosis for patients with HCC remains unfavorable, posing substantial challenges for clinical practitioners [4]. Therefore, the identification of predictive new targets associated with tumor and gaining a comprehensive understanding of the molecular genetic mechanisms underlying HCC tumor would greatly contribute to the overall clinical management of HCC patients.

The development of HCC is a multifactorial, multistage, and multigene process involving viral infections, oncogene activation, tumor suppressor gene inactivation, alterations in the tumor microenvironment, and metabolic reprogramming [5]. Among these factors, metabolic reprogramming, such as glucose metabolism, lipid metabolism, amino acid metabolism and so on, emerges as a noteworthy mechanism garnering substantial attention in the context of HCC proliferation, migration, and microenvironmental changes [5, 6]. Reports showed that lowered alanine levels have been found to promote non-adherent growth of tumor cells, while the modulation of metabolic processes can enhance their growth and survival [7]. The alanine has two isomers of α-alanine and β-alanine. β-alanine is a non-essential amino acid metabolized by carnosine, which plays an important role in cell metabolism, providing energy, regulating acid-base balance, and regulating protein synthesis [8]. Research has revealed that β-alanine possesses various anti-tumor effects, primarily achieved by diminishing cell migration and proliferation [9]. The formation of carnosine involves β-alanine and histidine binding together, resulting in the creation of an intracellular buffer with significant functionality [10]. In cancer, the formation of carnosine is considered an underdeveloped drug with potential therapeutic effects [8]. In particular, the buildup of carnosine has demonstrated the ability to inhibit the growth and proliferation of tumor cells cultured in the laboratory, as well as to suppress tumor growth in living organisms [11, 12].

Furthermore, to the best of our understanding, there is a notable scarcity of research exploring the relationship between β-alanine and HCC, particularly at the molecular level. Additionally, the identification of β-alanine-metabolism-related genes (βAMRGs) that could predict clinical outcomes and guide chemotherapeutic strategies in HCC patients remains unexplored. Hence, the potential development of an HCC risk stratification tool utilizing βAMRGs holds promise and warrants further investigation.

In our study, we undertook a comprehensive assessment of 22 βAMRGs in various cancer types, examining their expression levels and genomic variations. Leveraging the βAMRGs scores and expressions, we classified patients with HCC into three distinct subtypes, exploring their correlations with prognosis and immune microenvironments. Additionally, we devised and validated a novel, independent prognostic panel based on βAMRGs. To enhance the accuracy of HCC patients prognosis evaluation, we constructed a nomogram incorporating both the risk degree of the model and clinicopathological characteristics. These findings have significantly deepened our understanding of the pathophysiological mechanisms underlying HCC, providing valuable insights for clinical decision-making and fostering personalized treatment strategies. Moreover, our research highlights, for the first time, the crucial role of β-alanine in HCC, offering novel research targets and directions for the diagnosis and treatment of this condition. The findings of this study are anticipated to have a positive impact on the field, fostering advancements in our understanding and facilitating the development of enhanced strategies for managing HCC.

## MATERIALS AND METHODS

### Data collection and processing

To conduct this study, we obtained gene expression data and clinical annotations from two well-known databases: GEO and TCGA. To ensure the reliability of our analysis, we excluded patients without survival information or with survival times less than 30 days. Additionally, we used the ComBat method from the “SVA” package to address any batch effects caused by nonbiological technical biases [13]. The dataset for our study consisted of 569 HCC patients, incorporating information from TCGA-LIHC, GSE76427, GSE116174, and GSE144269 datasets. This comprehensive collection of expression profiles and clinical data enabled us to conduct a thorough and in-depth investigation of HCC. From the KEGG_BETA_ALANINE_METABOLISM dataset in the MsigDb platform, we extracted 22 classic βAMRGs.

The single-cell RNA (scRNA) sequencing data for HCC was sourced from the GSE166635 dataset [14], which included two HCC single-cell samples (GSM5076749 and GSM5076750). The Read10X function was employed to read the single-cell data, with the following filtering criteria set: min.cells = 3, min.features = 200, nCount_RNA ≥ 1000, nFeature_RNA ≥ 200, nFeature_RNA ≤ 9000, percent.mt ≤ 20.

### Pan-cancer analysis

To gain insights into the molecular features of βAMRGs in different human cancers, we conducted a thorough pan-cancer analysis, integrating genomic, transcriptomic, and clinical data [15, 16]. Our initial step involved gathering and consolidating raw data and clinical information from pan-cancer cohorts via the Xena website. The CNV and SNV data from the TCGA database were then processed and visualized as heatmaps, offering a comprehensive overview of variations in βAMRGs across various cancers. Furthermore, we conducted pan-cancer analyses on methylation levels and differential mRNA expressions.

To evaluate the prognostic significance of βAMRGs in diverse malignancies, we performed univariate COX regression analysis on their expression in relation to OS. By applying these extensive methodologies, our objective was to uncover the possible functions and importance of β-alanine metabolism-related genes in various cancer types, thus providing new perspectives and potential therapeutic avenues in the realm of cancer research.

### scRNA sequencing analysis

After single-cell quality control, we initially employed the FeatureScatter function to visualize the quality control results. The LogNormalize method was applied for data standardization [17], the FindVariableFeatures function for downstream dimensionality reduction analysis, the ScaleData function for data normalization, and the RunHarmony function for batch correction and dimensionality reduction analysis. The JackStraw function was utilized to determine the appropriate PC values, while the singleR function facilitated automated cell annotation. The AUCell, Add, singscore, ssGSEA, and UCell algorithms were employed to assess the metabolic states of each cell, and the cumulative results of all algorithm scores were referred to as ‘total scoring’ in this study, abbreviated as Scoring.

Violin plots were used to illustrate the differences in metabolic activity between each cell type. Finally, the CopyKat algorithm was employed to predict the benign or malignant nature of each cell. The Wilcoxon test function was used to compare metabolic differences between benign and malignant cells.

### Clustering analysis

Due to significant variations in gene expression profiles across the collected datasets, we devised a β-alanine metabolism score model to emphasize the distinct expression levels among samples. Initially, we employed the ssGSEA algorithm to calculate β-alanine metabolism enrichment scores for all 569 patients, thus assigning each patient a specific β-alanine metabolism score indicative of pathway activity.

Next, we conducted differential analysis, enabling us to explore expression pattern differences among the samples. These results were visually represented in a heatmap, generated using the “pheatmap” package, providing a comprehensive illustration of the clustering analysis outcomes.

By comparing mRNA expression levels of genes in tumor tissues with those in normal tissues, we successfully categorized mRNA expression statuses in tumor tissues into three distinct groups. This approach provided valuable insights into the dynamic nature of β-alanine metabolism across various cancer types, potentially revealing implications for cancer research and therapeutic strategies.

### Prognostic performances and molecular characteristics of β-alanine metabolism subtypes

To delve deeper into the connections between gene expression levels within these three clusters, we skillfully created violin plots using the “ggpubr” package to illustrate the enrichment scores among them. To examine prognostic differences among these clusters, we employed the “survival” and “survminer” packages in R.

By leveraging the resources of the MsigDb platform and drawing insights from previous research, we pinpointed 42 conventional metabolic pathways, 24 immune-related pathways, and 10 cell death pathways. To gauge the activity of these pathways within each subtype of β-alanine metabolism, we employed the GSVA program to calculate the metabolic score, immunological score, and cell death score for each liver cancer sample.

To assess variations in pathway activity among the three subtypes, we carried out the Kruskal-Wallis test. Furthermore, a comprehensive investigation was conducted to explore alterations in immune cell infiltration and expression of immune checkpoint genes (ICGs), providing detailed insights into the changes within the immune environment across diverse subtypes. For this analysis, we utilized various immunological algorithms available on the TIMER2.0 platform, including TIMER, CIBERSOFT, QUANTISEQ, EPIC, and others [18, 19]. Moreover, we applied the Kruskal-Wallis test to investigate variations in immune cell infiltration and expression of ICGs across the subtypes. We exclusively showcased the outcomes that demonstrated statistical significance with a P-value lower than 0.05.

### β-alanine metabolism is an auxiliary clinical indicator to predict the prognosis of HCC patients

The TCGA dataset contained 343 HCC samples, which were randomly divided into two groups: the training cohort, comprising 60% of the individuals, and the test1 cohort, including 40% of the participants. Moreover, all samples from the TCGA dataset were assigned to the test2 cohort (n=343), while the test3 cohort (n=226) exclusively comprised samples from the GEO platform.

To address collinearity and prevent overfitting in the model, Least Absolute Shrinkage and Selection Operator (LASSO) regression analysis was conducted on the 22 variables. Following that, multivariate Cox proportional hazards regression analysis was applied to compute risk scores for β-alanine metabolism using the “predict” function in R. Subsequently, the samples were divided into high-risk and low-risk subgroups based on the median risk score of the train cohort. Survival analysis using the Kaplan-Meier method was then employed on the train, test1, test2, and test3 cohorts to assess the predictive capacity of the risk scores.

To ascertain the independent prognostic value of the risk score, both univariate and multivariate Cox regression analyses were executed. Only indicators that demonstrated statistical significance (p<0.05) in both analyses were considered as independent prognostic factors. Given the variations in clinical information between the TCGA and GEO databases, independent prognostic analyses were solely conducted in the TCGA cohort. To provide a quantitative approach for predicting the survival probability of HCC patients in clinical practice, we employed the “rms” package in R to construct a nomogram plot. This plot integrated independent prognostic indicators based on the TCGA cohort. Furthermore, we conducted a calibration plot to assess the alignment between actual and nomogram-estimated survival probabilities. Additionally, ROC curves were utilized to verify the diagnostic performance in predicting 1-, 3-, and 5-year survival rates.

### Clinical significance and expression experimental verification of the EHHADH gene

The EHHADH gene constitutes a vital component of our HCC prognosis model. We have identified a significant correlation between this gene and various clinical-pathological indicators associated with HCC. As a result, our study exclusively delved into a thorough and comprehensive analysis, coupled with experimental validation of EHHADH. The findings presented in this study are predominantly derived from the BEST online platform, an open and encompassing multi-omics data visualization platform for diverse cancer types. Through queries involving HCC and EHHADH, we uncovered correlations between the gene and several key clinical indicators, including expression levels, age, grade, stage, BMI index, sorafenib sensitivity, microvascular invasion, among others. Furthermore, we conducted Kaplain-Meier survival analysis on multiple publicly available datasets to underscore the prognostic significance of EHHADH.

Utilizing the liver cancer tissue microarray (ZL-LivHcc962) procured from Shanghai Zhuoli Biotech Co., Ltd. (Zhuoli Biotech Co., Ltd., Shanghai, China), we conducted an analysis of EHHADH expression in both tumor tissues and their corresponding non-tumor counterparts. The tissue microarray underwent a 60-minute incubation in a constant temperature oven set at 59° C, followed by dewaxing and hydration using xylene and ethanol. Antigen retrieval was achieved through high-temperature and high-pressure treatment with EDTA. A 5% BSA blocking solution was administered dropwise and allowed to incubate at room temperature for 35 minutes. Subsequently, an optimal quantity of EHHADH antibody (Affinity Biosciences, DF4280, USA, 1:50) was introduced dropwise and incubated overnight at 4° C. The tissue microarray was then extracted, subjected to PBS washing, and exposed to a dropwise addition of the secondary antibody for a 35-minute incubation at 37° C. Diaminoaniline staining was employed for observation, followed by counterstaining with hematoxylin. The histochemistry score was computed based on the total area percentage and staining intensity, represented by the H-Score = ∑(pi×i) = (percentage of weak intensity×1) + (percentage of moderate intensity×2) + (percentage of strong intensity×3). Here, pi denotes the positive signal pixel area/cell count percentage, and i signifies the staining intensity. The H-score, ranging from 0 to 300, reflects a higher value corresponding to an augmented overall positive intensity of EHHADH.

### Availability of data and materials

The datasets examined in this study are available in the Supplementary Materials or can be obtained by contacting the corresponding author.

## RESULTS

### Pan-cancer analysis of 22 βAMRGs

**Figure 1:**
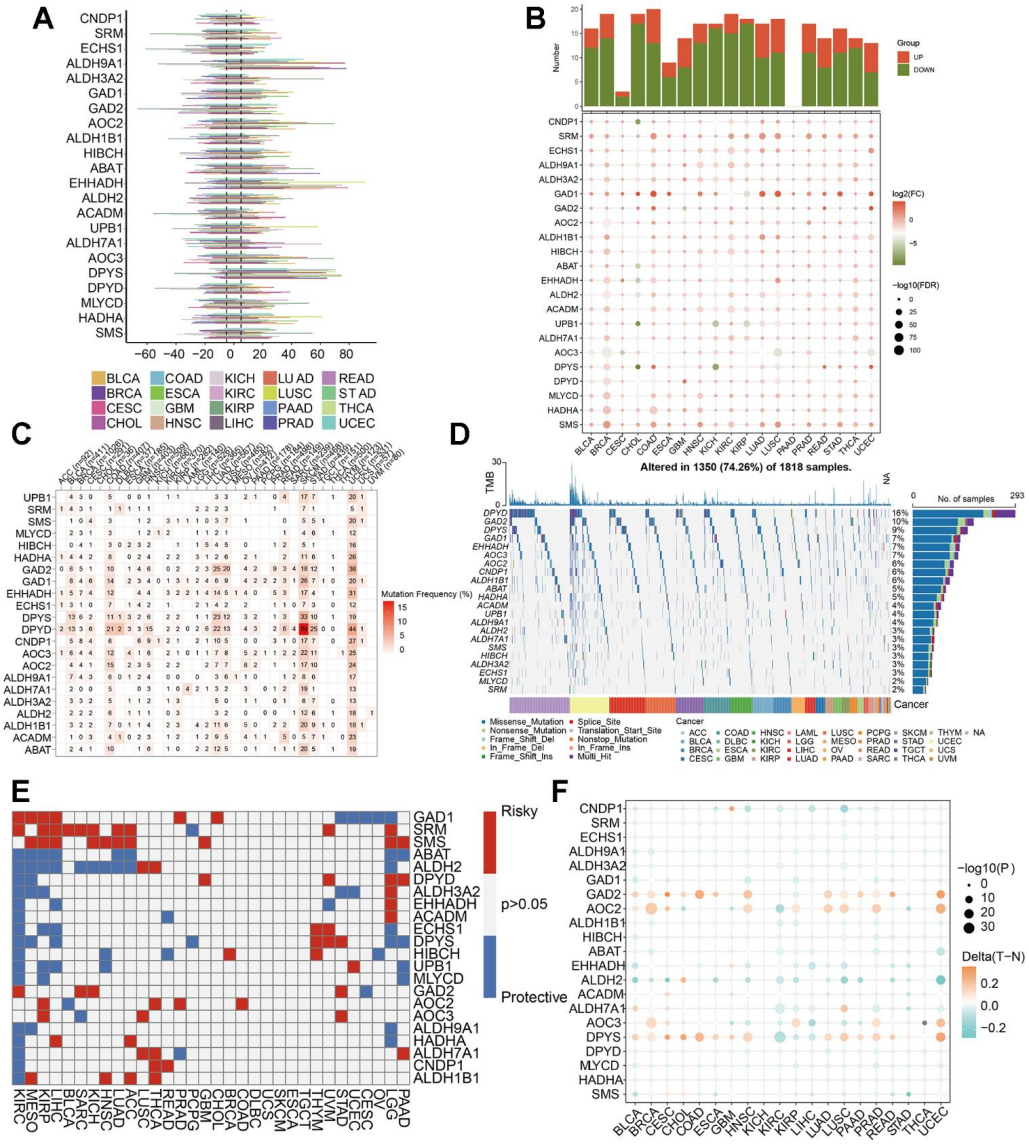
**Pan-cancer analysis of 22 βAMRGs.** (**A**) The frequency of CNV gains and losses was analyzed for the 22 βAMRGs across 20 different types of human cancers. The length of the line represents the variation frequency of the βAMRGs in pan-cancers. (**B**) The expression characteristics of the 22 βAMRGs were examined in 20 different types of human cancers, showing statistical significance (P < 0.05). (**C**, **D**) Heatmap and waterfall diagram were utilized to depict the SNV data of the 22 βAMRGs in pan-cancers. (**E**) Survival landscape analysis was conducted for the 22 βAMRGs across 20 different types of human cancers. Genes with P > 0.05 are represented by the white color, while red and blue colors indicate risk and protective genes, respectively. (**F**) The DNA methylation patterns of the 22 βAMRGs were examined in 20 different types of human cancers. The color gradient from orange to green signifies high to low methylation levels.

In order to explore the molecular characteristics of the 22 βAMRGs in diverse human cancers, comprehensive pan-cancer analyses were conducted, utilizing genomics, transcriptomics, and clinical data. Initially, we examined the mutational patterns of the 22 βAMRGs in human cancers and studied the prevalence of copy number variations (CNV). The results demonstrated that CNV occurred at a high frequency (ranging from approximately 10% to 80%) in various cancer types (Figure 1A). We also found the CNDP1 has a lower CNV frequency than other genes in human tumors. Then we researched the expression level of 22 βAMRGs in human tumors (Figure 1B). A significant number of the 22 βAMRGs exhibited differential expression between tumor tissues and adjacent normal samples in various human cancers. Furthermore, in nearly all human tumor tissues, the majority of βAMRGs were upregulated when compared to their expression levels in paired normal samples, particularly in cases of BRCA. We also found the SRM, GAD1 and SMS were significantly elevated in contrast with those in paired normal samples in human cancers. Next, we delved into the analysis of SNV within βAMRGs. Notably, BLCA, COAD, LUAD, LUSC, SKCM, STAD, and UCEC displayed a higher frequency of SNV, particularly in SKCM and UCEC (Figure 1C, 1D). Conversely, SNV frequency in other human tumors was relatively low, including ACC, CHOL, KICH, MESO, PCPG, TGCT, THYM, and UVM (Figure 1C, 1D). Remarkably, we observed that DPYD exhibited significantly higher SNV frequencies in human cancers, especially in SKCM, compared to other βAMRGs. These observations were visually illustrated through the heatmap and waterfall diagram of SNV (Figure 1C, 1D).

Subsequently, we explored the relationship of 22 βAMRGs expression with patient survival time (Figure 1E). By conducting a univariate Cox regression analysis, we identified βAMRGs that functioned as risk factors and those with a protective role (HR<1 and p<0.05). For instance, the most of the βAMRGs were the protective factors in the KIRC. It was worthy noticed that the ABAT βAMRG was the protective factors for human cancer. Methylation is a significant modification of proteins and nucleic acids, regulating gene expression and gene silencing. This epigenetic process plays a crucial role in various diseases, including cancer, aging, and Alzheimer’s disease, making it a vital focus of research in the field of epigenetics. DNA methylation is responsible for silencing specific genes, whereas demethylation leads to the reactivation and expression of these genes [20]. Hence, we researched that methylation patterns of βAMRGs in human cancer. According to the result (Figure 1F), the βAMRGs had complicated methylation patterns. For instance, the GAD2, AOC2 and DPYS had the hypermethylation in more than most of human cancers; on the contrary, most of βAMRGs showed the hypomethylation in more than most of human cancers. Interestingly, for LIHC, all the 22 βAMRGs showed the hypomethylation in human tumors.

### Single-cell analysis of two HCC patients

**Figure 2:**
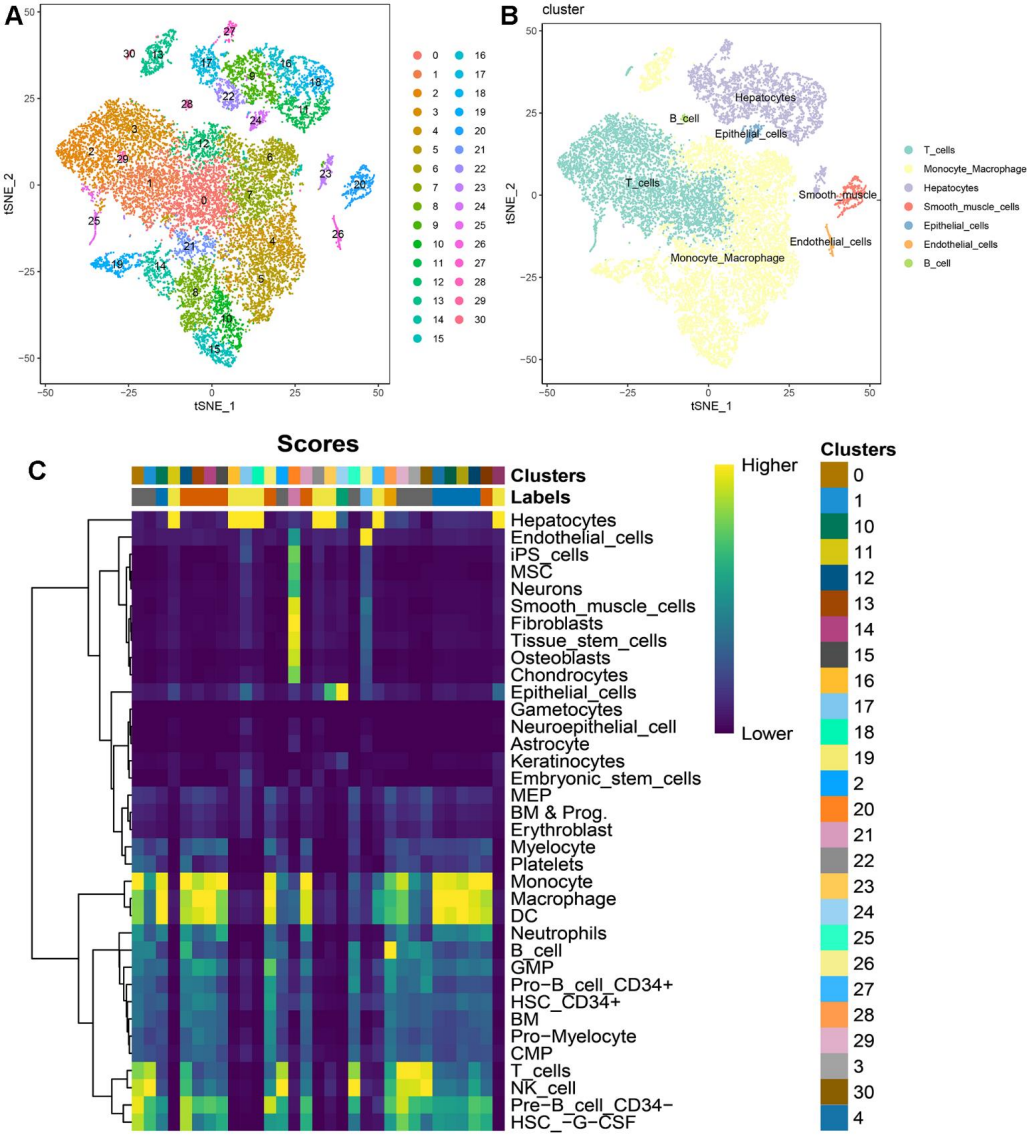
**Single-cell atlas of 2 HCC patients.** (**A**) t-SNE dimensionality reduction subtypes; (**B**) t-SNE dimensionality reduction cell annotations; (**C**) singleR automated annotation results.

According to the pre-established data quality control parameters, the single-cell quality control results for these two HCC patients were illustrated in Supplementary Figure 1A. The robust correlation between ncount_RNA and nfeature_RNA attested to the high quality of this batch of single-cell data (Supplementary Figure 1B). Notably, IGKC and IGHG1 genes emerged as significantly variable genes in this dataset (Supplementary Figure 1C). The single-cell atlas, both before and after batch correction using harmony, was presented in Supplementary Figure 1D, 1E. Overall, this study identified 31 cellular clusters (Figure 2A). Through automated annotation using singleR, seven distinct cell subgroups were conclusively recognized, encompassing T cells, B cells, monocytes, hepatocytes, smooth muscle cells, epithelial cells, and endothelial cells (Figure 2B, 2C).

**Figure 3:**
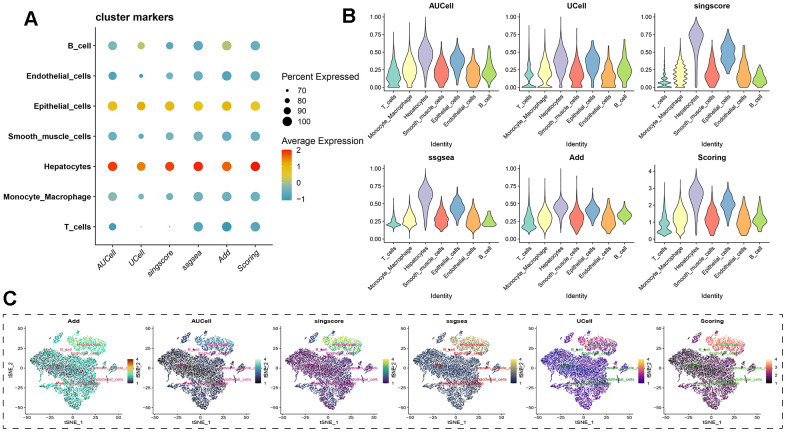
**Assessing beta-alanine metabolism at the single-cell level.** (**A**) Evaluation of the activation level of beta-alanine metabolism in each cell using six prediction algorithms; (**B**) Violin plots illustrating the variation in cellular activity levels of beta-alanine metabolism; (**C**) t-SNE plots depicting the dimensional distribution characteristics of beta-alanine metabolism.

**Figure 4:**
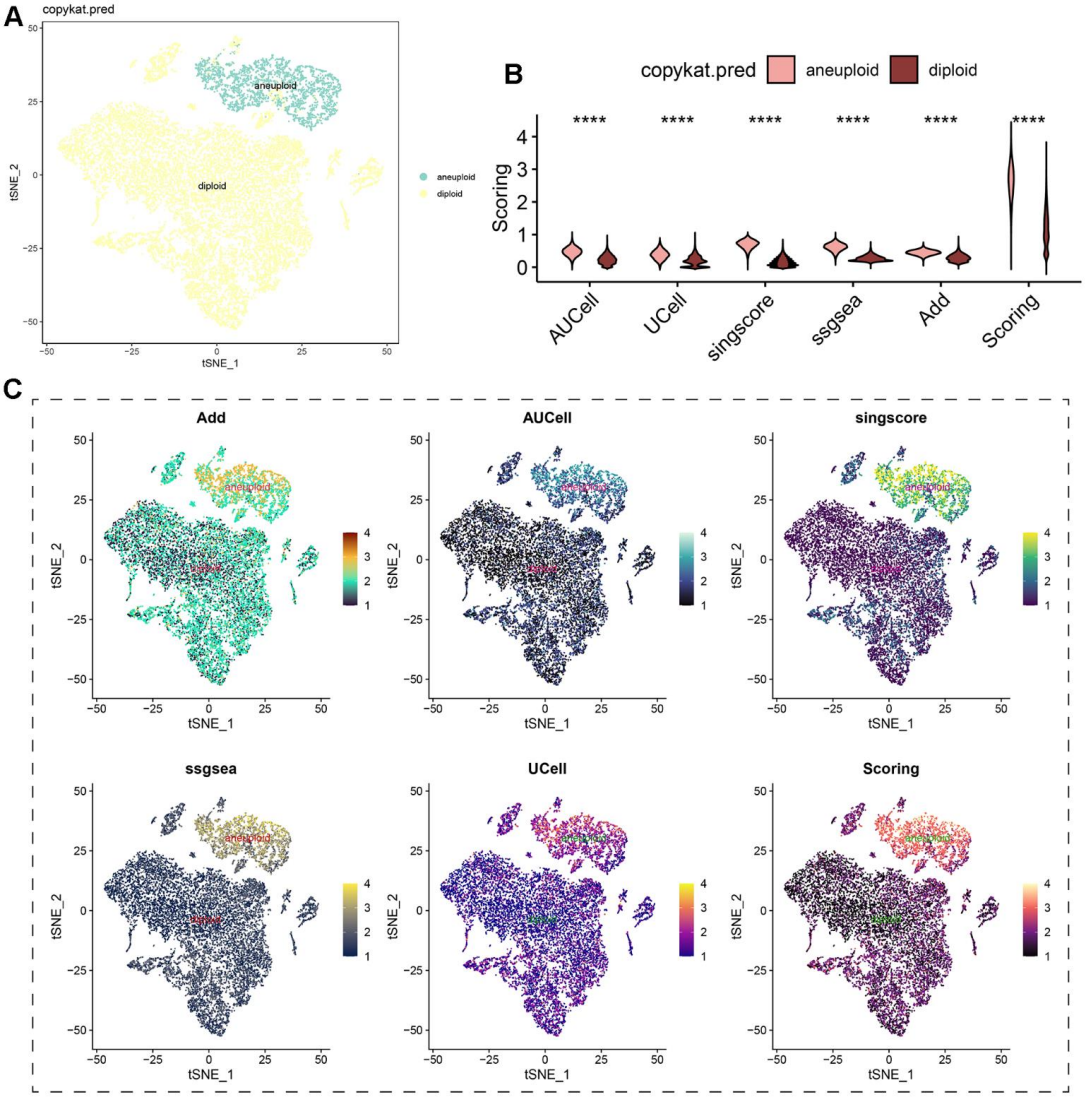
**Analysis of beta-alanine metabolism activity between benign and malignant cells.** (**A**) t-SNE dimensional distribution characteristics of benign and malignant cells; (**B**) Differences in the activity of beta-alanine metabolism between benign and malignant cells; (**C**) t-SNE plots illustrating the distribution characteristics of beta-alanine metabolism between benign and malignant cells.

Regardless of the prediction algorithm employed, consistently obtained results indicate that beta-alanine metabolism was predominantly active in hepatocytes and epithelial cells (Figure 3A, 3B). The dimensionality reduction maps of beta-alanine metabolism features for each cell were depicted in Figure 3C, highlighting that the region housing hepatocyte clusters exhibited higher beta-alanine metabolism scores. To assess differences in beta-alanine metabolism between benign and malignant cells, the CopyKat algorithm was utilized for prediction (Supplementary Figure 2). As depicted in Figure 4A, the majority of hepatocytes were classified as malignant cells, while other cell types were identified as benign cells. This alignment with actual data suggests relatively accurate and credible predictions. Quantitative analysis results indicated elevated levels of beta-alanine metabolism in malignant cells (Figure 4B). The t-SNE dimensionality reduction map further elucidated that the region housing malignant cells exhibited heightened beta-alanine metabolism activity (Figure 4C). In conclusion, all findings consistently supported the study’s conclusions.

### Cluster analysis for 343 HCC patients according to the βAMRGs scores

**Figure 5:**
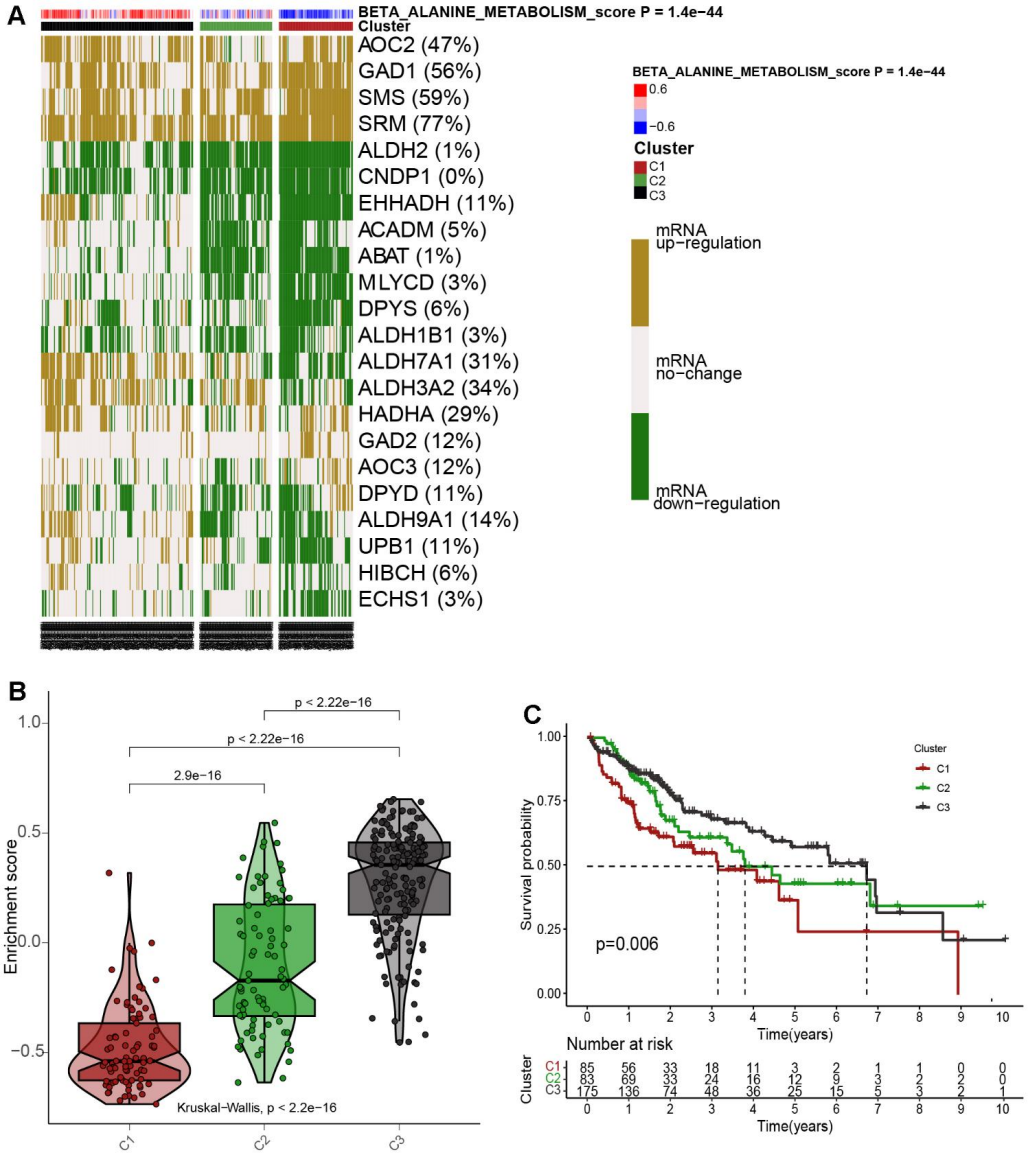
**Cluster analysis for 343 HCC patients according to the βAMRGs scores.** (**A**) The clustering of gene data reveals three distinct clusters, as depicted by the heatmap: Cluster 1(C1), Cluster 2 (C2), and Cluster 3 (C3), based on the levels of mRNA expression of βAMRGs. Cluster 2 (C2) comprises tumor patients with normal βAMRG expression, Cluster 3 (C3) includes those with active βAMRGs, while Cluster 1(C1) consists of individuals with inactive βAMRGs. (**B**) The violin plot illustrates the enrichment scores for the three clusters, arranged in the order of C3 > C2 > C1. The corresponding p-values are indicated above the respective clusters. (**C**) A survival curve represents the three distinct clusters. Cluster 3 exhibits the highest survival rate, while Cluster 1 demonstrates the lowest survival rate when compared among the three clusters. The x-coordinate represents survival time, while the y-coordinate represents survival rate.

Taking into account the impacts of βAMRGs in HCC, we initially assessed the enrichment scores of βAMRGs using ssGSEA for a total of 343 patients. These HCC samples were then categorized into three distinct clusters (Cluster1, Cluster C2, and Cluster C3) based on the mRNA expression levels of βAMRGs (Figure 5A). Specifically, the clusters named C1, C2, and C3 comprised patients with inactive, normal, and active βAMRGs, respectively. The violin plot illustrated that C3 exhibited the highest enrichment score, while C1 had the lowest, followed by C2 (Figure 5B). Subsequently, we investigated the relationship between the three clusters and overall survival by plotting the survival curves. Among the clusters, C3 displayed the highest overall survival rates, whereas C1 exhibited the lowest (Figure 5C). Additionally, the overall survival rates for HCC patients in C2 were found to lie between those of C1 and C3, indicating that high βAMRGs scores acted as a protective indicator. The β-alanine metabolism played an essential role in the outcome of HCC, particularly in cases of high activity of β-alanine metabolism. In summary, based on these results, we conclude that the classification technique for HCC is accurate, reliable, and scientifically sound.

### Correlations of the βAMRGs scores with tumor related metabolic, immune and cell death pathways in the 3 clusters

**Figure 6:**
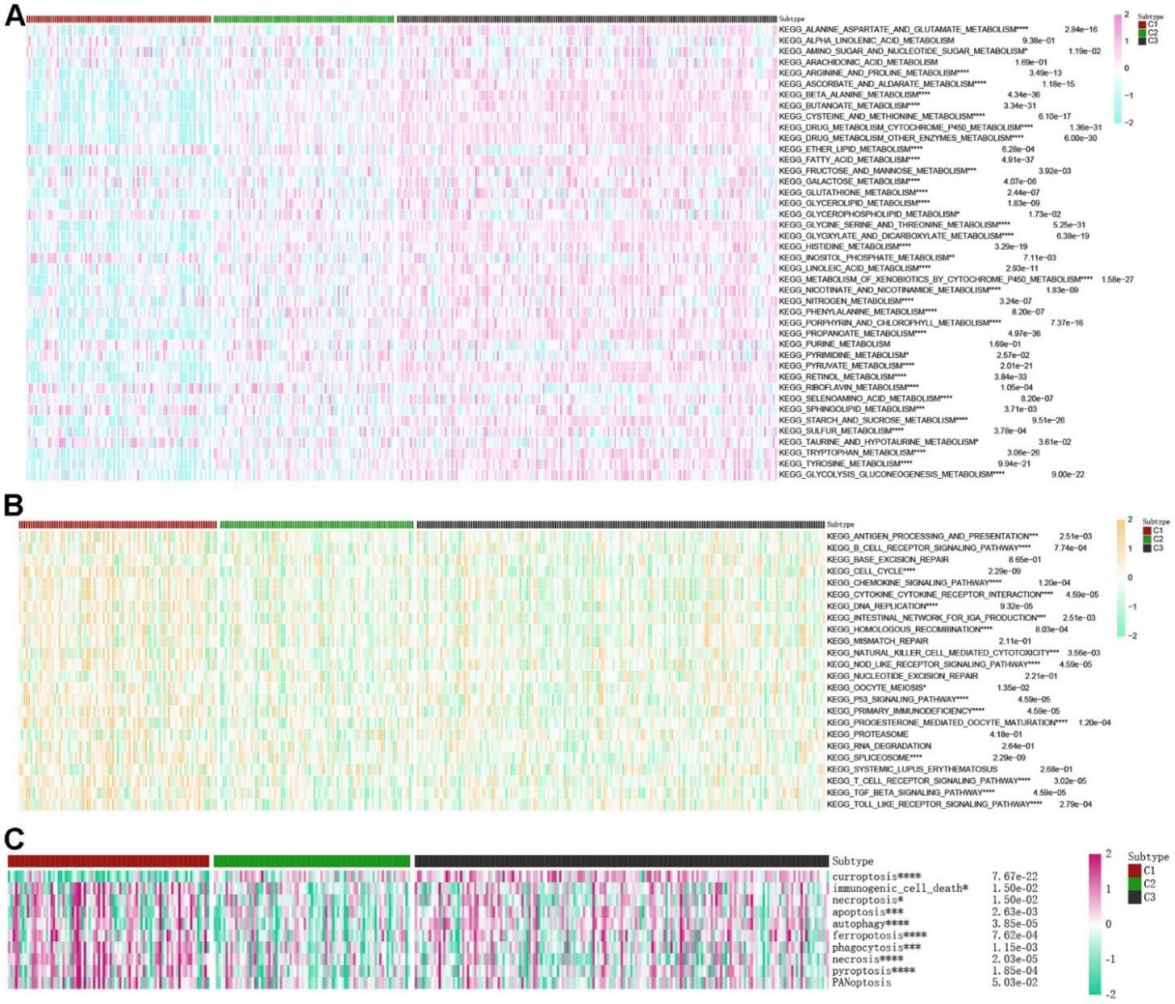
**Correlations of the scores of βAMRGs with tumor-related metabolic, immune, and cell death pathways in the three clusters were examined.** (**A**) The activity levels of 42 conventional metabolic pathways were analyzed across the three clusters. (**B**) The activity levels of 24 immune-related pathways were assessed across the three clusters. (**C**) The activity levels of 10 cell death pathways were investigated across the three clusters. (* indicates p <0.05; ** indicates p < 0.01; *** indicates p < 0.001; **** indicates p < 0.0001).

An analysis using a heatmap was conducted to examine the activity of 42 conventional metabolic pathways within the three clusters of βAMRGs (Figure 6A). Significantly different activity levels were observed across most of the 42 pathways in the three clusters. Notably, the C3 cluster exhibited the highest activities of various metabolism pathways, including alanine aspartate and glutamate metabolism, beta alanine metabolism, and glycolysis/gluconeogenesis metabolism. Conversely, a few metabolic pathways such as ether lipid metabolism, riboflavin metabolism, and inositol phosphate metabolism exhibited the lowest activity in the C3 cluster. These findings aligned with our expectations, as β-alanine metabolism plays both positive and negative roles in tumor-related metabolic pathways. Additionally, the activity of 24 immune-related pathways across the three clusters was explored (Figure 6B). Interestingly, nearly all immune-associated pathways displayed greater activity in the C1 cluster compared to C2 and C3 clusters, including pathways such as B cell receptor signaling pathway, natural killer cell mediated cytotoxicity, and T cell receptor signaling pathway. This observation suggests that HCC malignancy is highest in the C1 cluster, or that patients within C1 exhibit immune escape, resulting in a poorer prognosis. Next, the activity of 10 cell death pathways was examined in the three clusters (Figure 6C). Significant differences were observed in nine out of the ten cell death pathways, with the exception of the PANoptosis cell death pathway. Notably, a positive correlation was observed between curroptosis and β-alanine activity.

### Correlations between the βAMRGs scores and the immune cell infiltration in the 3 clusters

**Figure 7:**
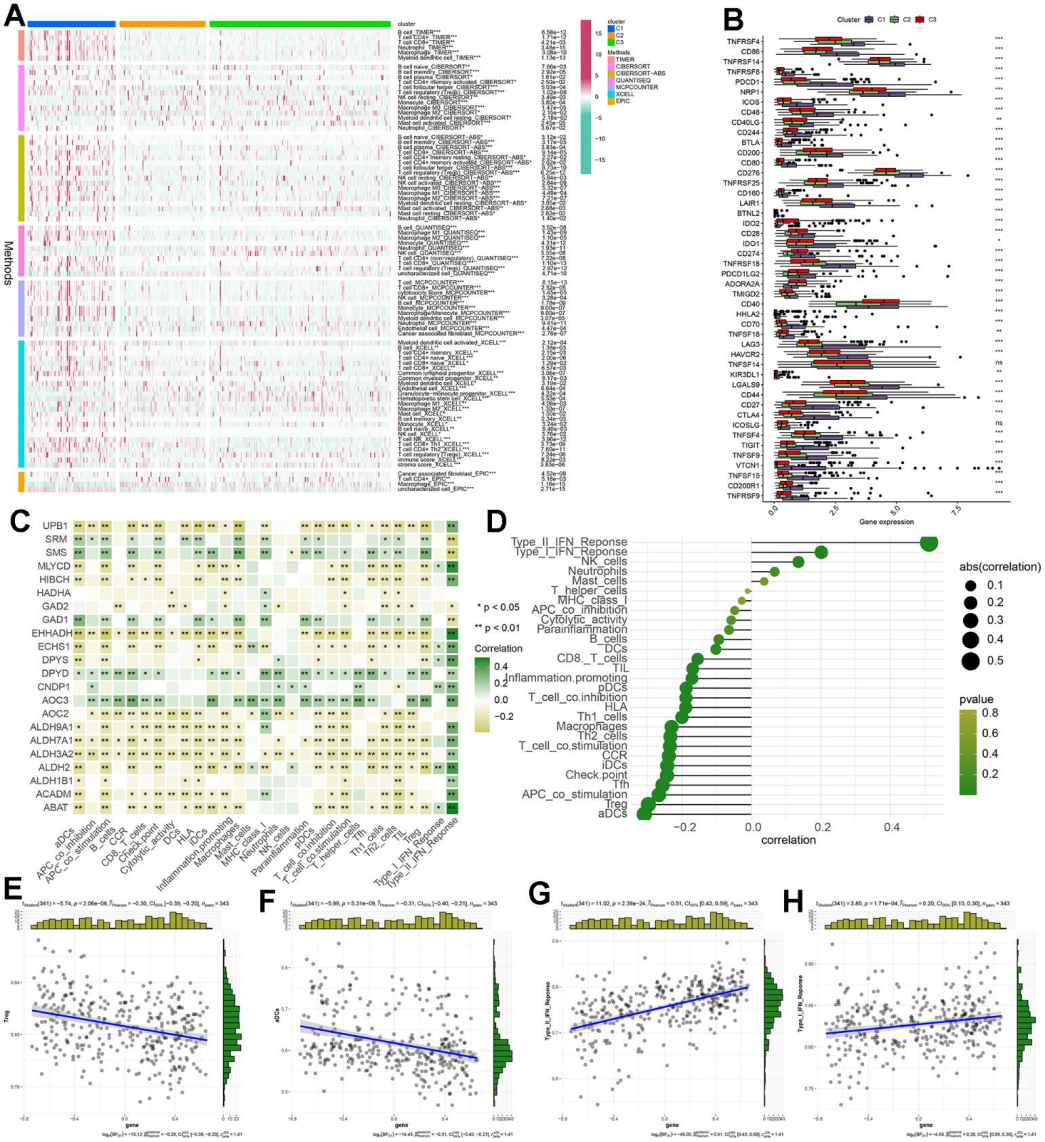
**The correlation between βAMRGs scores and immune cell infiltration within the three clusters was investigated.** (**A**) The heatmap displays variations in immune cell infiltration levels across the three subgroups based on βAMRGs scores. (**B**) The box plot demonstrates discrepancies in immune checkpoint expression among the three subgroups. (**C**) The heatmap depicts the relationship between βAMRGs-associated genes and immune cell infiltration levels. (**D**) The plot shows the link between βAMRGs scores and immune cell infiltration levels. The size of the sphere on the right side represents the correlation strength (abs) and the color represents the corresponding p-value. (**E**–**H**) The scatter plot depicts the relationship of βAMRGs scores with 4 immune-infiltration-related substances. The βAMRGs scores were shown to have a positive link to the infiltration levels of Type I IFN Response and Type II IFN Response. The βAMRGs scores were shown to have a negative link to the infiltration levels of aDCs and Treg. (* indicates p <0.05; ** indicates p < 0.01; *** indicates p < 0.001; **** indicates p < 0.0001).

The tumor microenvironment (TME) encompasses a complex interplay among cells, extracellular matrix, and signaling molecules in the vicinity of tumor cells [21]. In the context of hepatocellular carcinoma (HCC), the TME assumes a critical role as an intrinsic factor contributing to the initiation, progression, invasion, and metastasis of the disease [22]. Particularly relevant to the pathogenesis of HCC, the TME serves as a pivotal regulator by delivering, inhibiting, or promoting growth signals. Consequently, it represents a valuable resource for identifying potential therapeutic targets, particularly pertaining to tumor-infiltrating immune cells (TIICs) [23]. And our comprehensive review of the existing literature did not reveal any prior investigations on the association between β-alanine and TIICs. In order to depict variations in immune cell infiltration levels among the C1, C2, and C3 subgroups, a heatmap was generated using TIMER, CIBERSORT, CIBERSORT−ABS, QUANTISEQ, MCPCOUNTER, XCELL, and EPIC algorithms (Figure 7A). The heatmap illustrating immune cell infiltration demonstrated that most immune cells exhibited the highest infiltration levels in the C1 cluster and the lowest levels in the C3 cluster. These results suggest that βAMRGs scores play a crucial role in modifying immune cell infiltration in HCC. Immune checkpoints are a molecular signaling pathway that regulates the immune response to maintain the balance of the immune system and prevent excessive activation. However, tumor cells can use these immune checkpoints to evade attack by the immune system, thereby promoting tumor growth and spread [24]. Hence, we studied the expression level of immune checkpoints related genes (ICGs) in different expression of βAMRGs clusters (Figure 7B). Our findings revealed a correlation between the βAMRGs-inactive cluster and the overexpression of immune checkpoint genes (ICGs), indicating that patients in the βAMRGs-inactive group exhibited the weakest anti-tumor immunity and poor prognostic outcomes.

Our study also endeavors to elucidate the relationship between βAMRGs’s levels and immune microenvironment (Figure 7C). We observed a predominantly negative correlation between the majority of βAMRGs (e.g., UPB1, EHHADH, ALDH9A1, ALDH7A1) and immunocyte infiltration, as well as immune-related functions. Conversely, βAMRGs such as SRM, SMS, GAD1, DPYD, and AOC3 exhibited a positive association with the levels of immune cell infiltration. And with the exception of SRM, SMS, GAD2, and GAD1, all βAMRGs exerted a positive influence on the Type II IFN Response. Finally, we evaluated the relationship between βAMRGs scores and immune cell infiltration (Figure 7D–7H). Notably, mast cells, neutrophils, NK cells, Type I IFN Response, and Type II IFN Response demonstrated a positive correlation with βAMRGs scores, while the remaining infiltrating immune cells exhibited a negative correlation, especially for aDCs and Treg.

### Construction and verification of a novel βAMRGs related prognostic signature(βAMRGs-RPS) for predicting the clinical outcomes of patients with HCC

**Figure 8:**
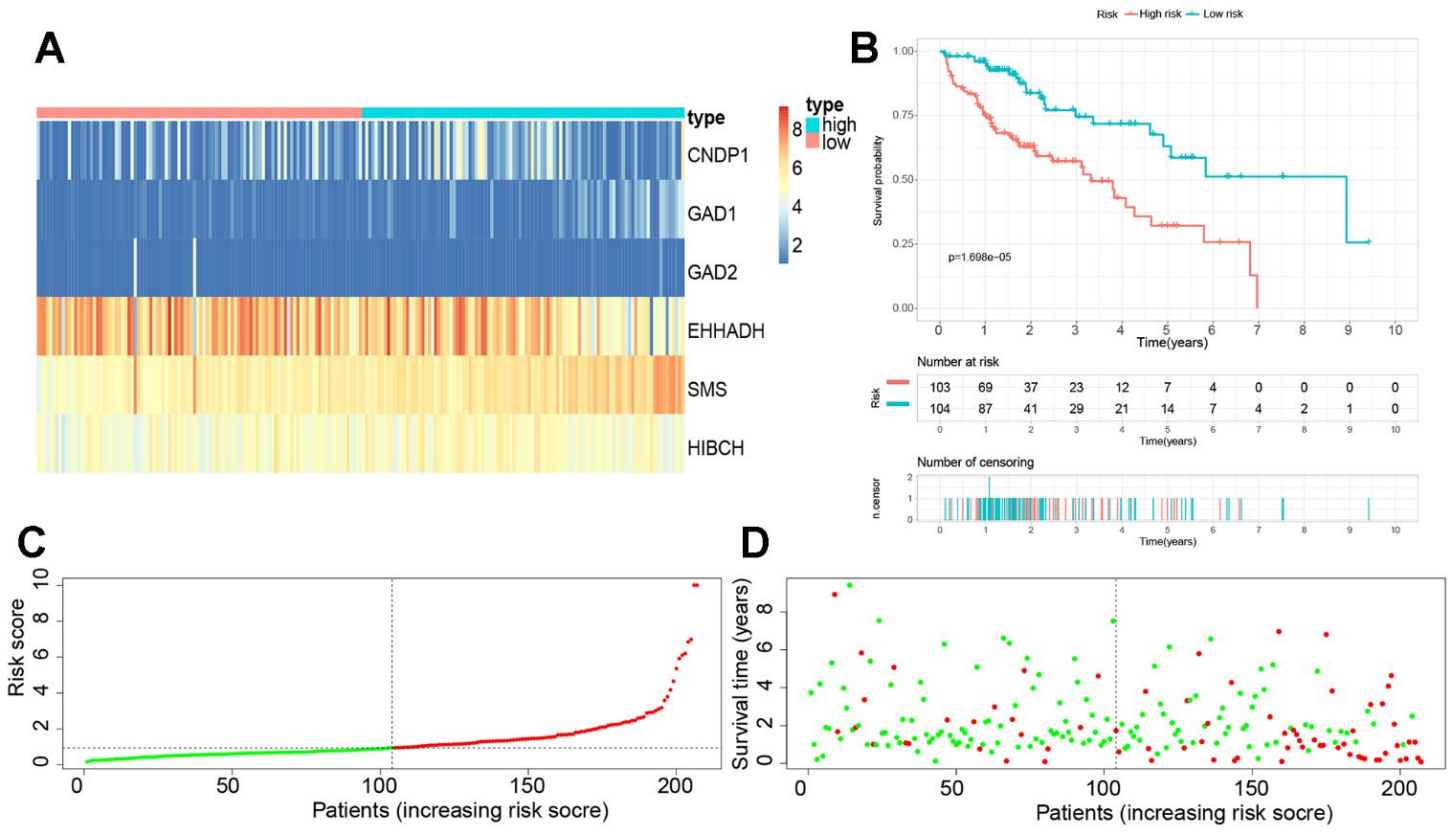
**Establishment of a novel βAMRGs-RPS in the train cohort.** (**A**) Heatmap illustrating the expression levels of six genes between subgroups with high and low risk scores. (**B**) Prognostic prediction depicted by the Kaplan-Meier survival curve comparing subgroups with high and low risk scores. (**C**) Risk score curve plot exhibiting the distribution of individual risk scores, with the patients categorized into low-risk (green) and high-risk (red) groups. (**D**) Risk score scatter plot, where red dots indicate deceased patients and green dots indicate surviving patients.

A total of 22 βAMRGs from previous studies were utilized for the LASSO-Cox regression analysis. Subsequently, seven out of the 22 βAMRGs were selected for further multivariate Cox regression analysis (Supplementary Figure 3A, 3B). Finally, a multivariate Cox proportional hazards regression analysis was conducted, integrating six central hub βAMRGs (i.e., SMS, HIBCH, GAD1, GAD2, CNDP1, EHHADH), to construct the βAMRGs-RPS (Supplementary Figure 3C). In cohort 1, which comprised 343 TCGA samples, the three GEO cohorts were merged to form cohort 2. Cohort 1 samples were randomly divided in a 6:4 ratio, with 60% assigned to the training group, 40% to the test1 group, and all samples of TCGA assigned to the test2 group. The cohort 2 samples constituted the test3 group. Based on the median risk score, the patients in the training set were categorized into high- and low-risk groups. The expression levels of the six genes were visualized using a heatmap, depicting the differences between the high- and low-risk score groups (Figure 8A). The survival curve analysis revealed that the high-risk group had a poorer overall survival compared to the low-risk group (Figure 8B). Furthermore, the HCC patients were stratified into low- and high-risk subgroups based on the median risk score (Figure 8C). The high-risk subgroup exhibited a significantly higher mortality rate compared to the low-risk subgroup, as indicated by the risk score distributions and survival status (Figure 8D). These findings indicate that the βAMRGs-RPS can accurately distinguish HCC patients with a favorable or unfavorable prognosis.

To validate the reliability and stability of the βAMRGs-RPS, heatmaps were generated for the test1, test2, and test3 cohorts, demonstrating a similar expression pattern of the six βAMRGs as observed in the training cohort (Supplementary Figures 4A, 5A, 6A). Notably, both internal and external validation of the survival data demonstrated that HCC patients with low-risk scores exhibited significantly higher overall survival rates (p < 0.05) (Supplementary Figures 4B, 5B, 6B). Additionally, the HCC samples in the test1, test2, and test3 cohorts were also classified into low- and high-risk populations using the same bioinformatics methodology (Supplementary Figures 4C, 5C, 6C). It is important to note that the median risk score from the training cohort served as a consistent benchmark for separating HCC samples. The risk score distributions and survival status in the internal validation cohorts (test1 and test2) as well as the external validation cohort (test3) showed similar trends to those observed in the training cohort (Supplementary Figure 4D, 5D, 6D). These results highlight the reliability and accuracy of the βAMRGs-RPS.

### Constructing a nomogram plot by integrating independent prognostic indicators based on the TCGA cohort

**Figure 9:**
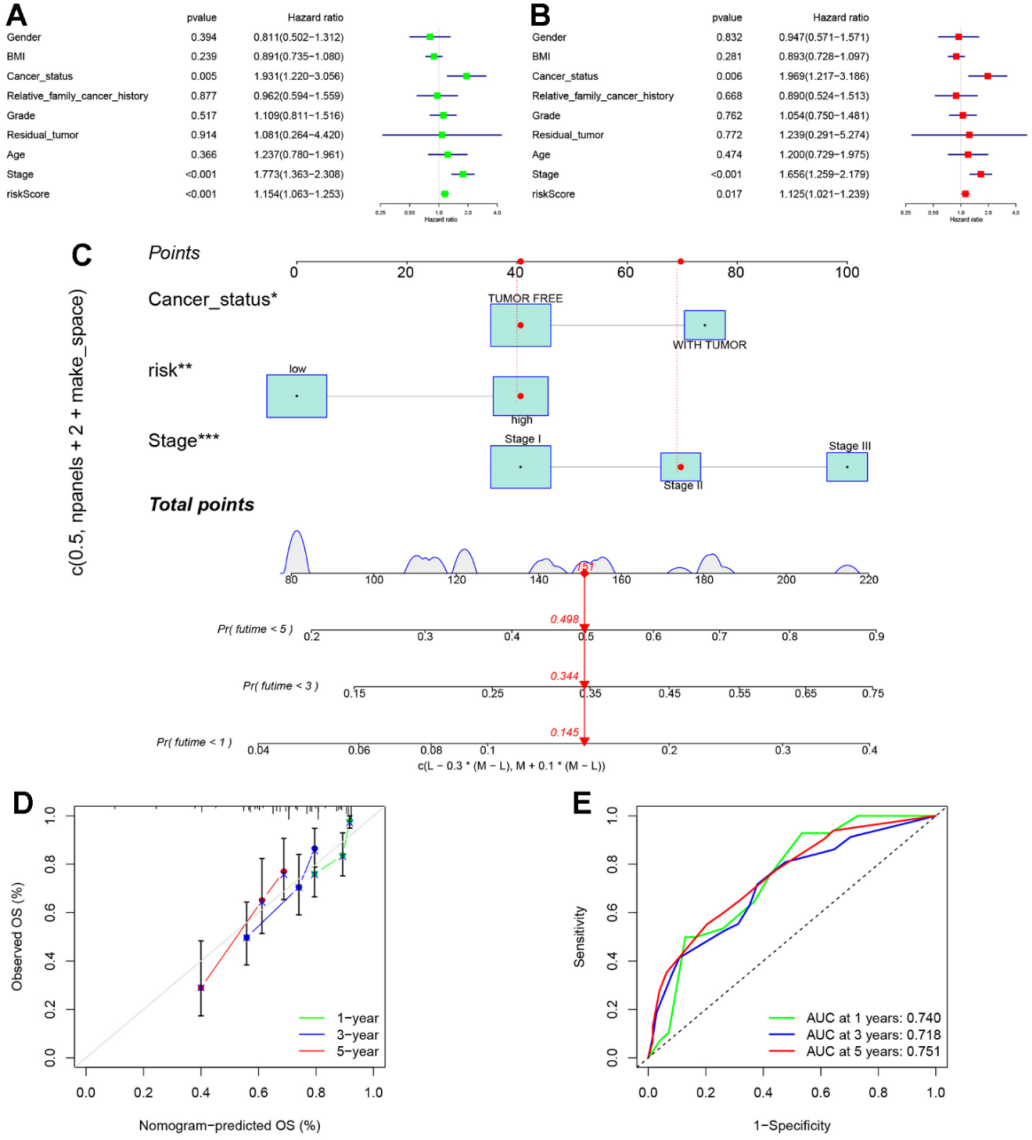
**Establishment of nomogram.** (**A**) Univariate analyses were conducted to assess the relationship between risk scores and relevant clinical parameters and OS in the TCGA cohort. (**B**) Multivariate analyses were performed to evaluate the relationship between risk scores, relevant clinical parameters, and OS in the TCGA cohort. (**C**) Development of a signature-based prognostic nomogram was undertaken to predict OS in HCC. (**D**) Calibration curves of the nomogram prediction of 1-year, 3-year, and 5-year OS of patients in TCGA cohort. (**E**) ROC curve of the risk score. The AUC values for the 1-, 3-, and 5-year ROC curves were found to be 0.740, 0.718, and 0.751, respectively.

To further investigate the precision of this signature, a comprehensive analysis was undertaken to evaluate its potential as an independent prognostic factor for overall survival (OS) within the TCGA cohort. Both univariate and multivariate analyses were conducted, incorporating the risk score and clinical traits as variables. The results of the univariable Cox regression analysis revealed significant correlations between OS and cancer status, stage, and the risk score (p < 0.05) (Figure 9A). Subsequently, the multivariate analysis confirmed cancer status, stage, and the risk score as independent predictors for OS (p < 0.05) (Figure 9B). Based on these three variables, a nomogram for OS was constructed (Figure 9C). The calibration curves demonstrated a high degree of concordance between the actual proportions of 1-, 3-, and 5-year OS and the predicted probabilities derived from the nomogram (Figure 9D). Furthermore, to assess the accuracy and stability of the nomogram, a receiver operating characteristic (ROC) curve analysis was performed. The AUC values for the 1-, 3-, and 5-year ROC curves were found to be 0.740, 0.718, and 0.751, respectively (Figure 9E). The ROC curve further substantiates the novelty, reliability, and practicality of our findings.

### Clinical significance and expression experimental verification of the EHHADH gene

**Figure 10:**
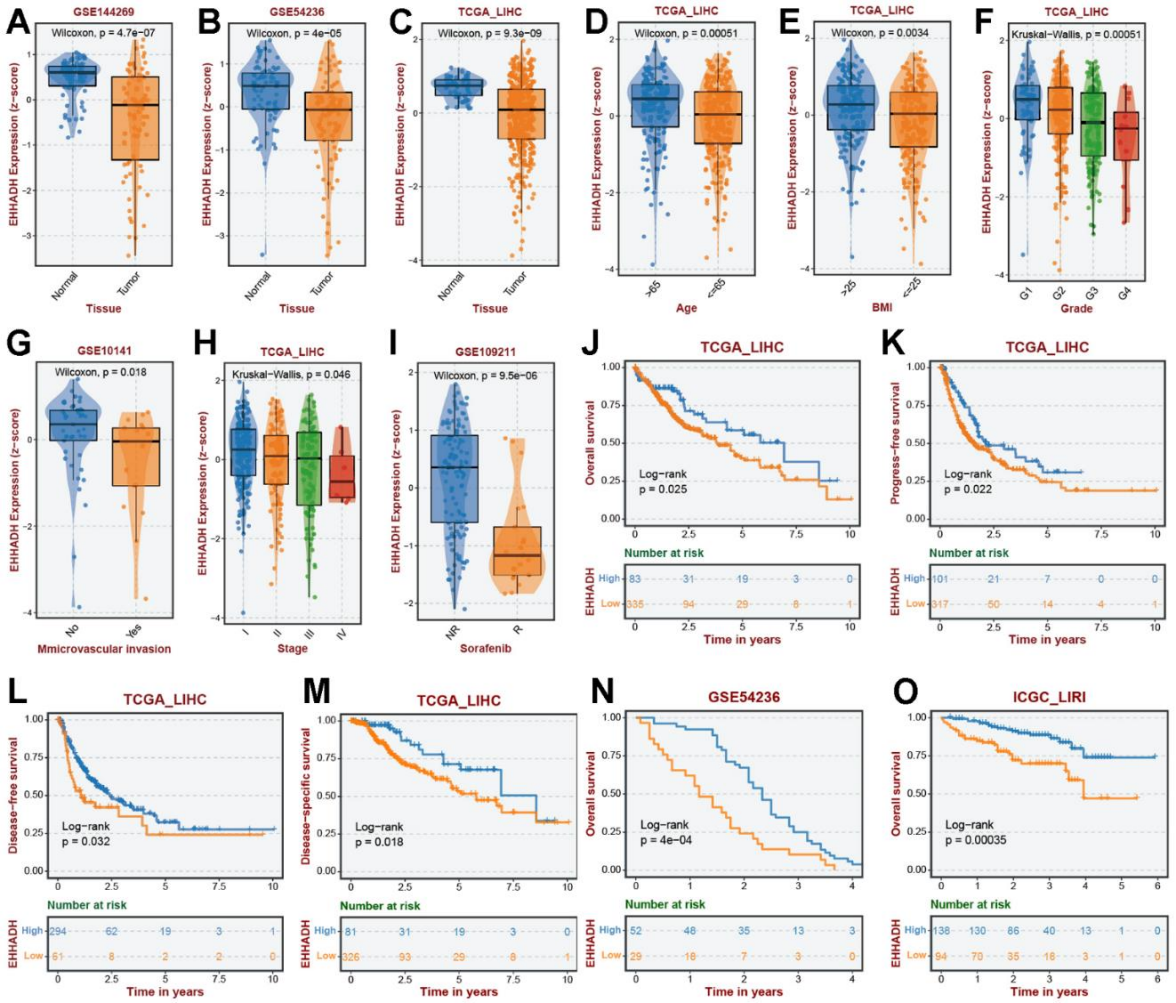
**Association of EHHADH expression with clinical traits.** The expression traits of EHHADH in (**A**) GSE144269, (**B**) GSE54236, (**C**) TCGA cohorts. Association of EHHADH expression with (**D**) age, (**E**) BMI, (**F**) grade, (**G**) microvascular invasion, (**H**) stage, and (**I**) sorafenib response. Prognostic performances of EHHADH in (**J**–**O**) multiple HCC cohorts.

**Figure 11:**
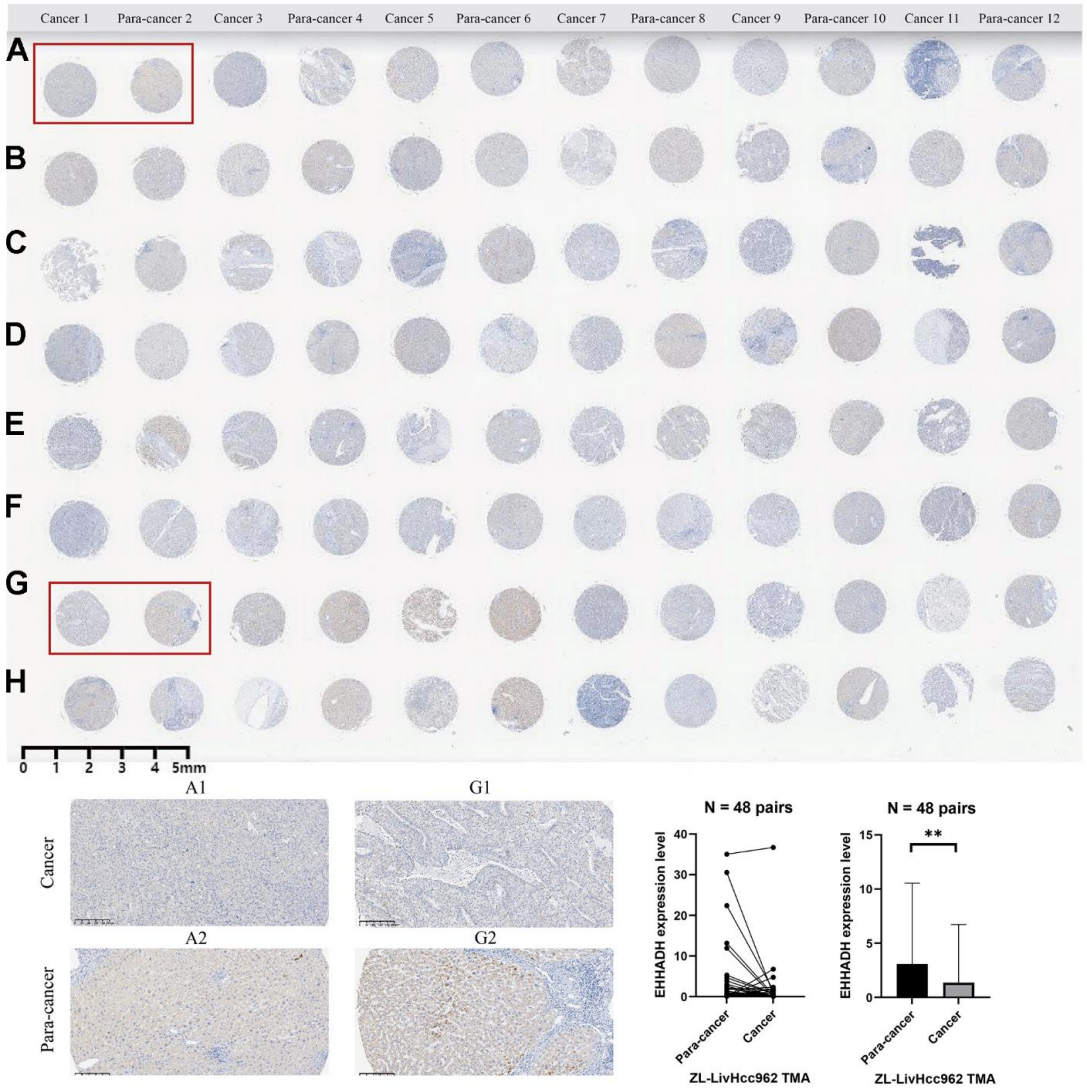
**Immunohistochemical experiment and statistical analysis of the EHHADH gene based on HCC tissue microarray.** (**A**–**H**) merely represent the rows of liver cancer tissue chips, without any special significance that needs to be reflected in the paper.

The EHHADH gene played a pivotal role in our prognostic model, and we have identified it as a potential protective protein in HCC. Our findings reveal a significant decrease in EHHADH gene expression in tumor tissues compared to adjacent non-cancerous tissues (Figure 10A–10C). Moreover, patients with a younger age and lower BMI index exhibit reduced levels of EHHADH gene expression (Figure 10D, 10E). As histological and pathological stages progress, the expression of the EHHADH gene further diminishes, strongly indicating its potential as a protective factor in HCC (Figure 10F–10I). Additionally, patients with microvascular invasion and those with a poor response to sorafenib treatment also exhibit lower levels of EHHADH gene expression (Figure 10F–10I). Decreased levels of EHHADH gene expression often correlate with a poorer prognosis for HCC patients (Figure 10J–10O), and targeting the activation of EHHADH may represent a viable strategy to prolong patients’ survival. Finally, we complemented the immunohistochemical experiments of EHHADH with a tissue microarray of liver cancer (Figure 11). The results illustrated a clear trend of downregulation in the protein levels of EHHADH in the majority of liver cancer samples.

## DISCUSSION

Beta-alanine, a non-essential amino acid, is endogenously synthesized through the degradation of carnosine, anserine, acrolein, and dihydropyrimidine. It constitutes a vital component of carnosine and is widely recognized as a key constituent in various sports supplements. Within the human body, carnosine, composed of beta-alanine and histidine, assumes the role of a natural antioxidant primarily in skeletal muscles [10]. We posit that the antioxidant properties of carnosine may not only enhance athletic performance but also contribute to anti-aging processes and fortify the immune system response by counteracting free radicals and mitigating oxidative stress. The inhibitory effect of beta-alanine on cancer cell proliferation and growth is attributed to the formation of L-carnosine [12, 25]. Previous investigations have documented that the synthesis of carnosine from beta-alanine and histidine effectively impedes cancer cell proliferation [25]. Furthermore, research has demonstrated the inhibitory impact of carnosine on human cervical tumor cells [26, 27]. Muthuraman Pandurangan1 proposes that beta-alanine could potentially serve as an antineoplastic agent, exhibiting diverse anticancer effects in renal and cervical tumor cells [28]. Additionally, earlier studies have established that beta-alanine-mediated inhibition of PTHR1 reduces the proliferation, invasion, migration, and tumorigenesis of osteosarcoma cells [29]. Nonetheless, investigations on the role of beta-alanine in tumor biology remain in the preliminary stage, with limited scientific literature available. The precise mechanisms and efficacy of beta-alanine in tumor treatment, particularly in HCC, as well as its contribution to the initiation and progression of HCC, have yet to be conclusively determined. Consequently, a promising and innovative approach for prognostic evaluation and personalized therapy in HCC involves the stratification of HCC risk based on beta-alanine.

Firstly, we conducted a comprehensive pan-cancer analysis of the 22 βAMRGs using bioinformatics-related methods, marking the first exploration of their involvement in various human tumors. Specifically, scRNA analysis revealed that beta-alanine metabolism was mainly activated in malignant hepatocytes. Furthermore, we examined mutation profiles, expression levels, prognostic implications, and methylation patterns of βAMRGs. Our results demonstrated that upregulation of βAMRGs can effectively impede cancer progression, corroborating earlier research findings [3, 9, 28, 29]. Moreover, our findings indicated that appropriate clinical treatment of HCC. However, HADHA, one of the βAMRGs, has been previously implicated in the upregulation of βAMRGs could potentially enhance prognoses in patients with HCC, thereby offering novel insights and avenues for investigating the mechanistic actions of βAMRGs in fundamental research and detrimental effects observed in HCC [30]. Furthermore, the HADHA has been implicated in fostering ovarian cancer progression through the up-regulation of CDK1 and is identified as a potential risk factor for malignant lymphoma [31, 32]. Moreover, the expression level of HADHA has been shown to intricately correlate with tumor advancement and prognosis in humans, with varying effects [31]. The aforementioned studies have elucidated that various βAMRGs exert distinct roles in human tumors, collectively influencing tumor prognosis and clinical outcomes. This phenomenon may stem from the inherent heterogeneity and individual variances within human tumors. In this thesis, our focus will be on elucidating the significant role of βAMRGs in HCC.

We then classified HCC patients into three clusters according to their βAMRGs expression. Our findings demonstrate the accurate and significant categorization of HCC patients into C1, C2, and C3 groups, each displaying distinct prognostic implications. Patients with HCC who belonged to the βAMRGs active cluster had a best survival rate compared to those belonging to the βAMRGs inactive cluster. Similar to the results of pan-cancer analysis, we provided further evidence of the significant role played by βAMRGs in the occurrence and progression of tumors. Thus, our stratification approach holds crucial value and significance in terms of prognostic evaluation and clinical guidance for patients with HCC.

As widely recognized, tumor-related metabolic and immune pathways play a crucial role in the prognosis and progression of human cancer [33, 34]. In recent years, the apoptotic pathway, specifically cell apoptosis, has emerged as a significant target for tumor drug development. However, due to tumor cells evading apoptosis, leading to treatment resistance and recurrence, extensive research has focused on alternative forms of tumor cell death, including necroptosis, pyroptosis, ferroptosis, and autophagy. These regulated cell death (RCD) mechanisms have been extensively studied and demonstrated to be essential for effective cancer treatment [35]. Consequently, in this study, we investigated the correlations between the scores of βAMRGs and tumor-related metabolic, immune, and ten cell death pathways within the C1, C2, and C3 clusters. As anticipated, significant differences were observed in the aforementioned pathways among the three clusters. Tumor-associated metabolic pathways exhibited higher activity in C3 compared to C1 and C2, whereas immune-associated pathways displayed decreased activity in C3 relative to C1 and C2. Interestingly, contrary to conventional belief associating active tumor-associated metabolism and inactive immune-associated pathways with poor prognosis, our findings revealed that HCC in C3 exhibited the most favorable prognosis. These contrasting results might be attributed to undiscovered pathways or potential regulation of the tumor microenvironment (TME) and immune checkpoint expression by βAMRGs, among other unknown mechanisms. Furthermore, we observed that βAMRGs were associated with nine cell death pathways, with cuproptosis significantly more active in C3 than in C1 and C2. High expression of cuproptosis-related genes, such as FDX and SLC31A1, has been reported to indicate a favorable prognosis in HCC [36]. Therefore, the regulation of cell death pathways by βAMRGs may have implications for the prognosis and development of HCC, providing a foundation and novel perspective for investigating the mechanisms underlying the action of βAMRGs in HCC.

The TME encompasses the intricate interplay of immune cells, tumor cells, stromal cells, and extracellular matrix surrounding the tumor. It exerts a significant regulatory influence on tumor growth, invasion, and metastasis. The infiltration of immune cells assumes a crucial role in shaping the tumor immune microenvironment, thereby influencing the prognosis of HCC and the response to immune checkpoints [37, 38]. Consequently, we investigated the relationship between immune cell infiltration factors and βAMRGs in our study. Our findings underscore the indispensable role of βAMRGs in immune cell infiltration, suggesting that the βAMRGs-inactive subgroup of HCC may exhibit a higher degree of malignancy or harbor immunosuppressive cells or immune escape and other phenomena. Notably, the elevated expression of certain immune checkpoints, including CTLA-4 and PDCD1, signifies a poor prognosis for HCC [37]. Our results confirm that most immune checkpoints (such as CTLA-4 and PDCD1) in the C3 subtype (βAMRGs-active) exhibit low expression levels, implying an augmented anti-tumor immune response. These observations substantiate the superior clinical outcomes observed in patients belonging to the C3 group, aligning with the conclusions drawn from our study. Moreover, immunosuppressive cells are known to exert their functions through various mechanisms, including regulatory T cells (Tregs), tolerogenic dendritic cells, and M2 macrophages, thereby suppressing the immune system. These cells have been associated with a dismal prognosis in HCC [38–40]. On the other hand, cancer-inhibiting cells such as Type I IFN Response and Type II IFN Response showed a positive correlation with βAMRGs scores [41]. In conclusion, our findings support the hypothesis that βAMRGs scores are associated with Tregs, tolerogenic dendritic cells, M2 macrophages, Type I IFN Response, and Type II IFN Response. The abundance of cancer-promoting immune cells mentioned above showed a negative correlation with βAMRGs scores, whereas cancer-inhibiting immune cells displayed a positive correlation. These observations may explain the poorer prognosis in the C1 and C2 subtypes compared to the C3 subtype in HCC.

Taking into account the aforementioned results, it is evident that βAMRGs play a significant role in the development of HCC. However, due to the molecular heterogeneity and complex functions of each βAMRG, the classification may not accurately predict the clinical outcome of each patient. As a result, we have developed a molecular prognostic signature that has the ability to accurately predict clinical outcomes in patients with HCC. To formulate this prognostic signature, we employed LASSO-Cox regression analysis, resulting in a set of six βAMRGs (SMS, GAD1, HIBCH, GAD2, CNDP1, and EHHADH). Previous studies have already reported on the expression levels and functions of these six hub genes in human tumors and HCC. Specifically, SMS (spermine synthase) has been identified as up-regulated in HCC, serving as a potential biomarker for poor prognosis in HCC patients. Additionally, the involvement of SMS in the tumor immune microenvironment suggests its potential as a target for HCC immunotherapy [42]. GAD1 (glutamate decarboxylase 1), on the other hand, performs the catalysis of the inhibitory neurotransmitter gamma-aminobutyric acid synthesis, utilizing pyridoxal 5’-phosphate as a cofactor. Studies have reported that GAD1 upregulation alters local glutamate metabolism in the brain metastasis microenvironment, promoting metabolic adaptation and facilitating brain metastasis growth. However, research on the function and mechanisms of GAD1 in HCC remains scarce, necessitating further investigation [43]. While there is a lack of research on the molecular characteristics of HIBCH (3-hydroxyisobutyryl-CoA hydrolase) in HCC, high expression of HIBCH has been linked to poor survival in colorectal cancer patients, as well as increased cell growth, resistance to apoptosis, and reduced autophagy in colorectal cancer cells. Targeted reprogramming of HIBCH is employed in the treatment of colorectal cancer valine metabolism [44]. Only one study has reported that CNDP1 may serve as a potential biomarker for diagnosing and evaluating the prognosis of HCC, exhibiting some complementarity with serum AFP [45]. Furthermore, EHHADH is associated with improved survival outcomes through the activation of immune checkpoints and exhibits significant downregulation in HCC tissues [46]. However, the role of GAD2 (glutamate decarboxylase 2) in HCC has not been explored. In summary, these 6 βAMRGs utilized in the establishment of the βAMRGs-RPS hold considerable significance in HCC. Additionally, our study lays the foundation for further investigations and offers new avenues for exploring the functions of these 6 βAMRGs.

Finally, the construction of a cancer prognosis nomogram carries significant value and importance in the field of bioinformatics analysis. Such a nomogram serves as an essential tool that provides predictive information concerning the prognosis of patients with HCC, thus aiding in the guidance of treatment decisions. Accordingly, we constructed a nomogram plot by integrating the cancer status, stage, and the risk score, based on data from the TCGA cohort. The ROC curve demonstrates that the nomogram can function independently as a prognostic marker for HCC, offering guidance for clinical decision-making and providing robust support for selecting appropriate treatment options for patients.

The study possesses several limitations that merit acknowledgement. Firstly, the precise mechanism through which the identified βAMRGs in our prognostic signature modulate the processes involved in HCC remains unclear. Therefore, it is imperative to conduct well-designed experiments to further explore and elucidate their biological function. Secondly, the development and validation of our model relied solely on retrospective data acquired from public databases. This reliance underscores the necessity for additional prospective studies to confirm the clinical utility of our findings. Finally, a comprehensive investigation into the biological function and expression levels of βAMRGs necessitates further exploration through meticulously designed experiments. Notwithstanding these limitations, it is essential to acknowledge the advantages and clinical significance of our findings. Our study sheds light on the critical role of βAMRGs in HCC and offers valuable insights for both basic research and clinical treatment pertaining to βAMRGs within the context of HCC. The nomogram model contributes to the understanding and management of HCC. However, it is imperative to address the aforementioned limitations through further studies and investigations in order to advance our knowledge in this field.

## CONCLUSIONS

In conclusion, this study thoroughly examined the molecular properties of βAMRGs and their prognostic potential in HCC. A prognostic signature was developed using six βAMRGs. By incorporating the risk scores, cancer status, and stage, we constructed a nomogram capable of predicting patients’ OS. Moreover, these findings suggest that the identified βAMRGs may serve as promising therapeutic targets in HCC. Yet, to unveil the underlying mechanisms by which these βAMRGs contribute to HCC progression, further experimental studies are essential.

## Supplementary Material

Supplementary Figures
