# Supplementary material for: Comprehensive characterization of β-alanine metabolism-related genes in HCC identified a novel prognostic signature related to clinical outcomes
Source: Aging (Albany NY). 2024 Apr 16;16(8):7073–100. doi: 10.18632/aging.205744 (PMC11087131; doi:10.18632/aging.205744)
Supplement: Supplementary Figures [file aging-16-205744-s001.pdf]

SUPPLEMENTARY FIGURES

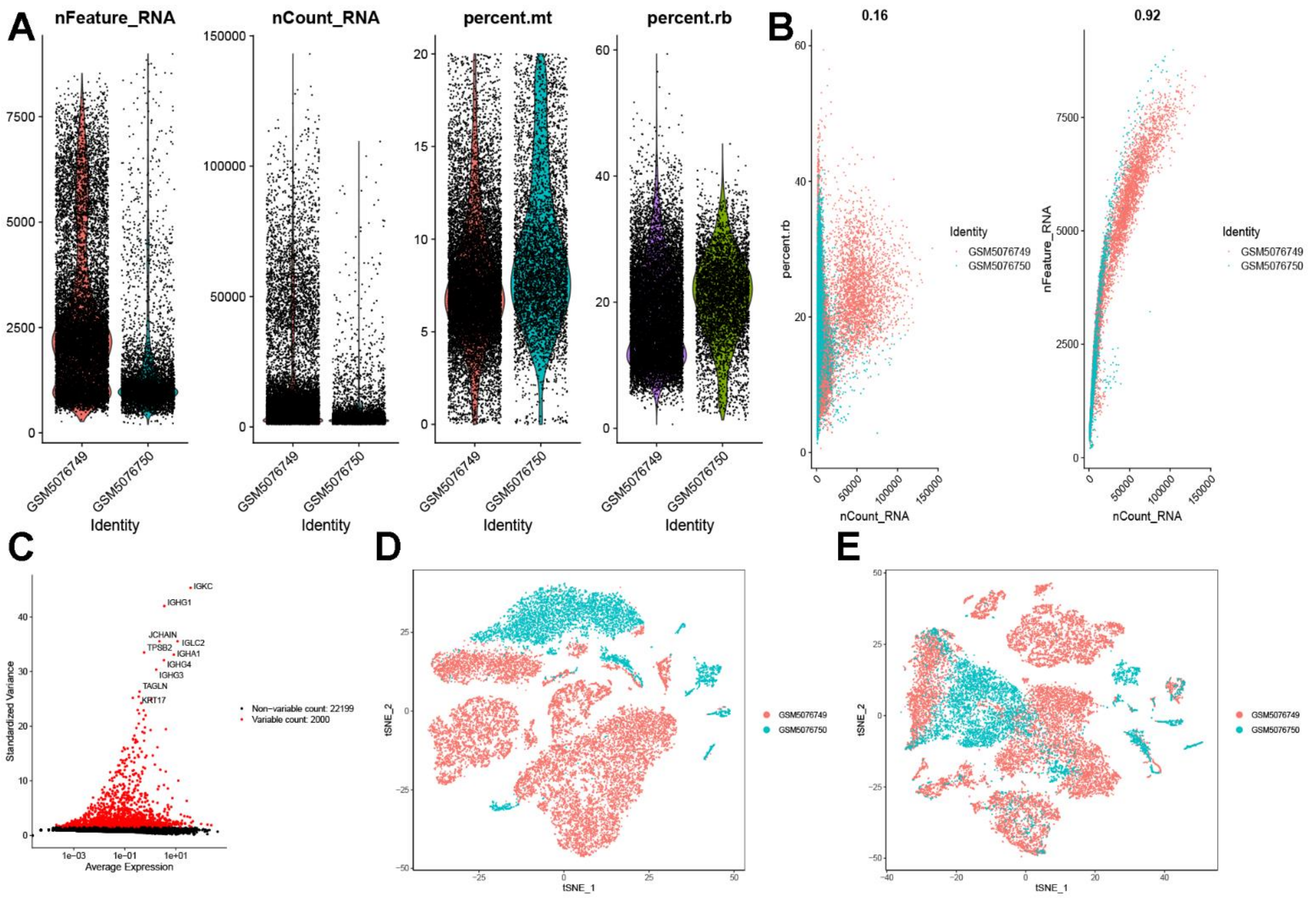

Supplementary Figure 1. Single-cell data processing steps. (**A**) By controlling the sequencing depth, number of genes, mitochondrial content and ribosome content, the unqualified cells are filtered out; (**B**) Correlation between above indicators; (**C**) Identification of highly variable genes; Batch correction and dimensionality reduction using Harmony before (**D**) and after (**E**) analysis.

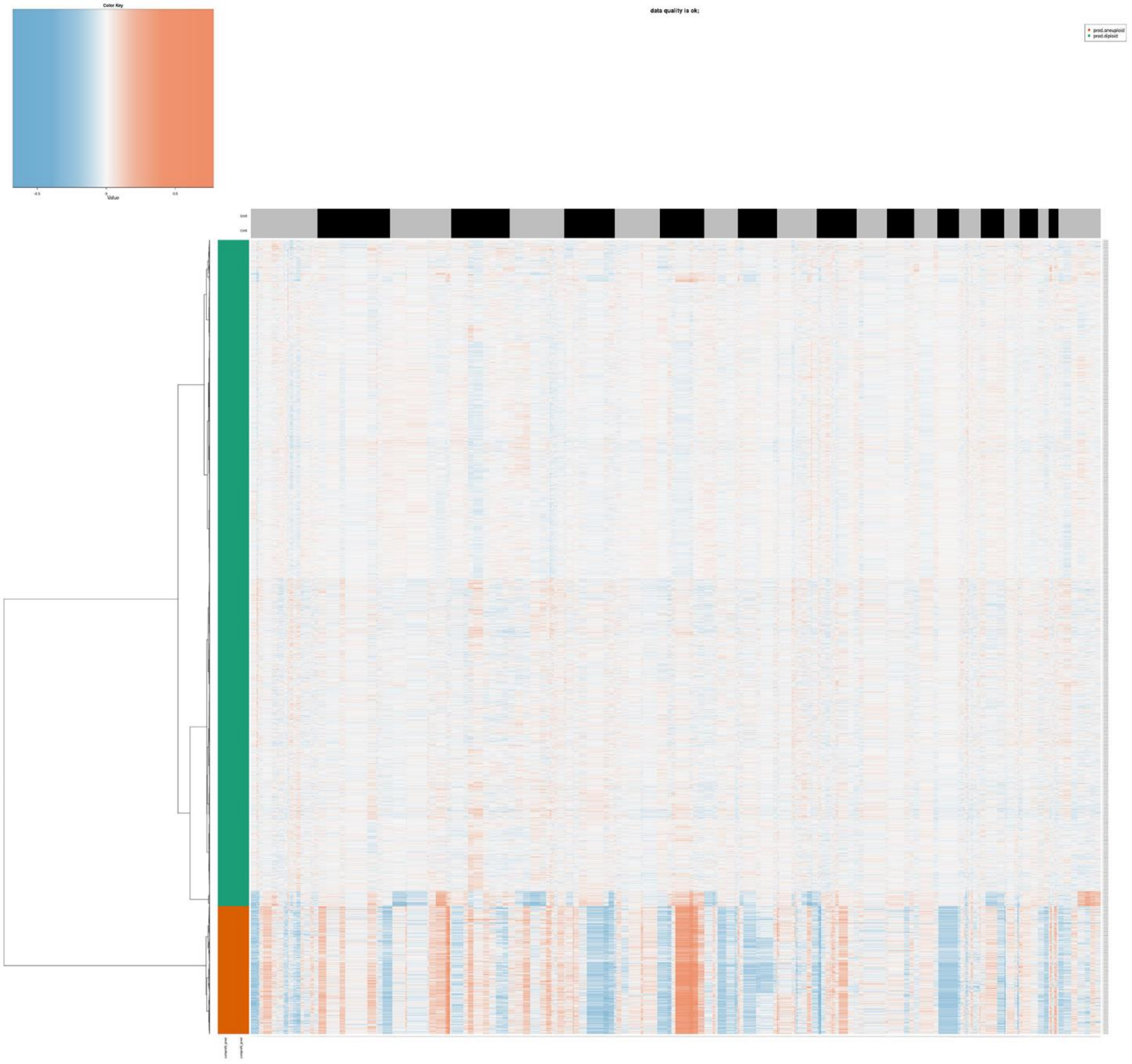

Supplementary Figure 2. The results of CopyKat algorithms.

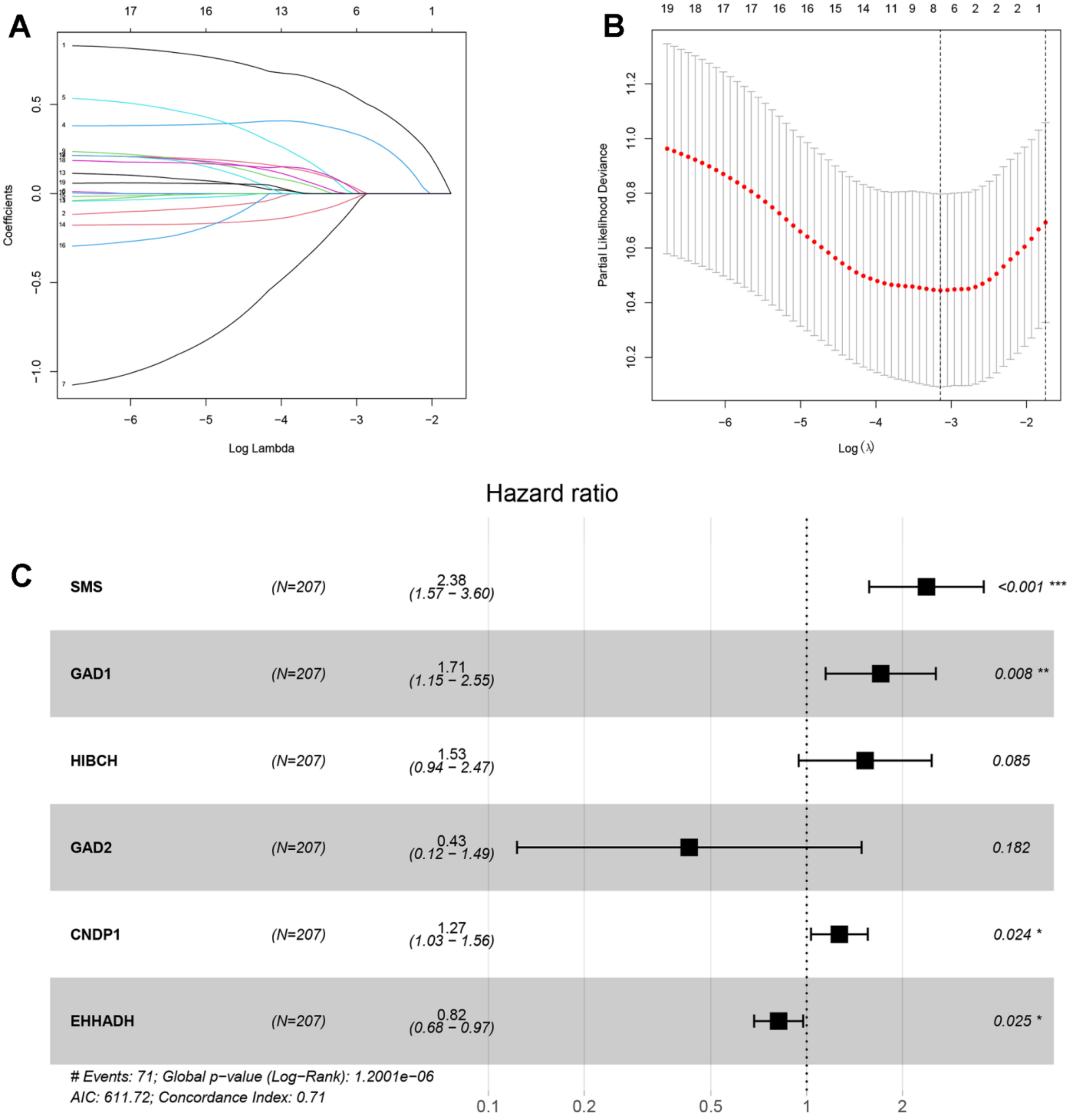

Supplementary Figure 3. Screening of the hub genes of βAMRGs-RPS. (**A**) LASSO coefficient profiles of βAMRGs mRNA in HCC. (**B**) Cross-validation results of the model construction. A total of six genes were selected using LASSO Cox regression analysis. (**C**) Analysis of multivariate Cox regression of βAMRGs mRNA.

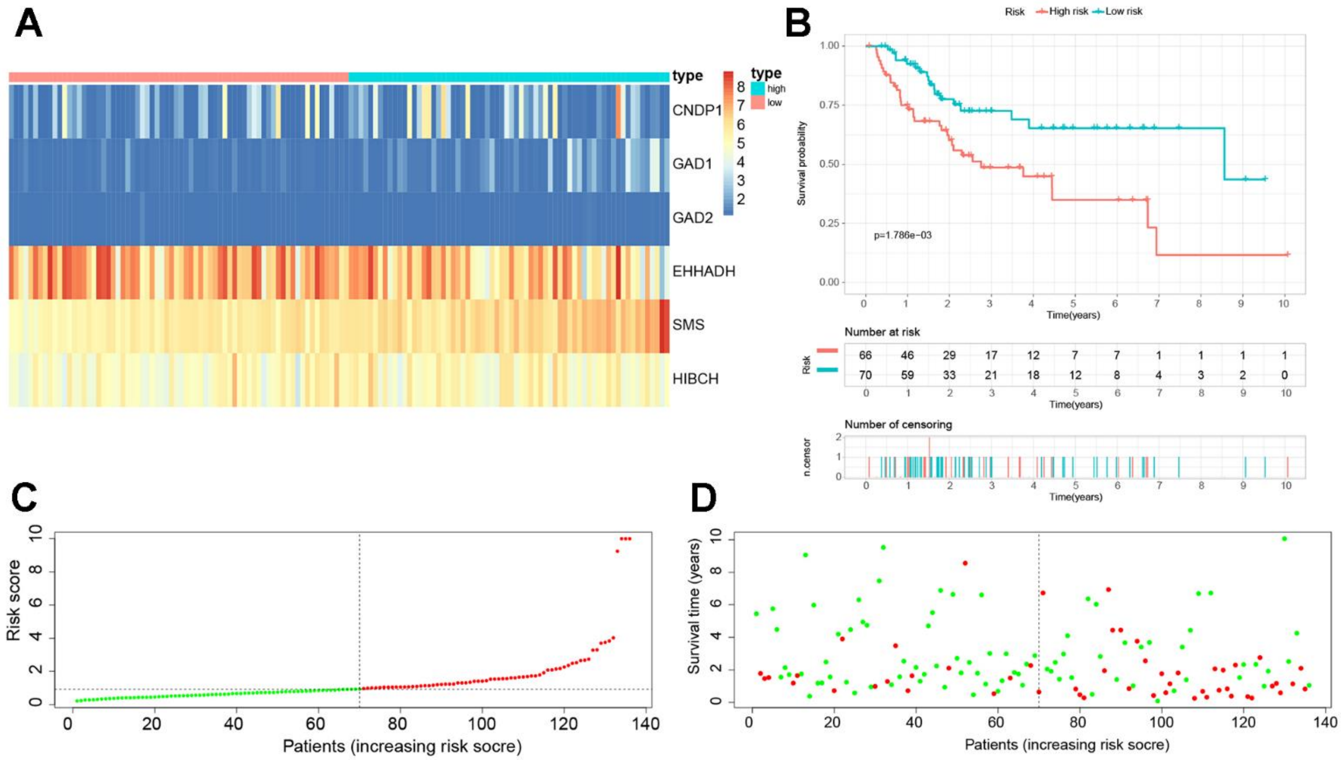

Supplementary Figure 4. Internal validation of the robust βAMRGs-RPS in the test1 cohort. (**A**) Heatmap illustrating the expression levels of six genes between subgroups with high and low risk scores. (**B**) Prognostic prediction depicted by the Kaplan-Meier survival curve comparing subgroups with high and low risk scores. (**C**) Risk score curve plot exhibiting the distribution of individual risk scores, with the patients categorized into low-risk (green) and high-risk (red) groups. (**D**) Risk score scatter plot, where red dots indicate deceased patients and green dots indicate surviving patients.

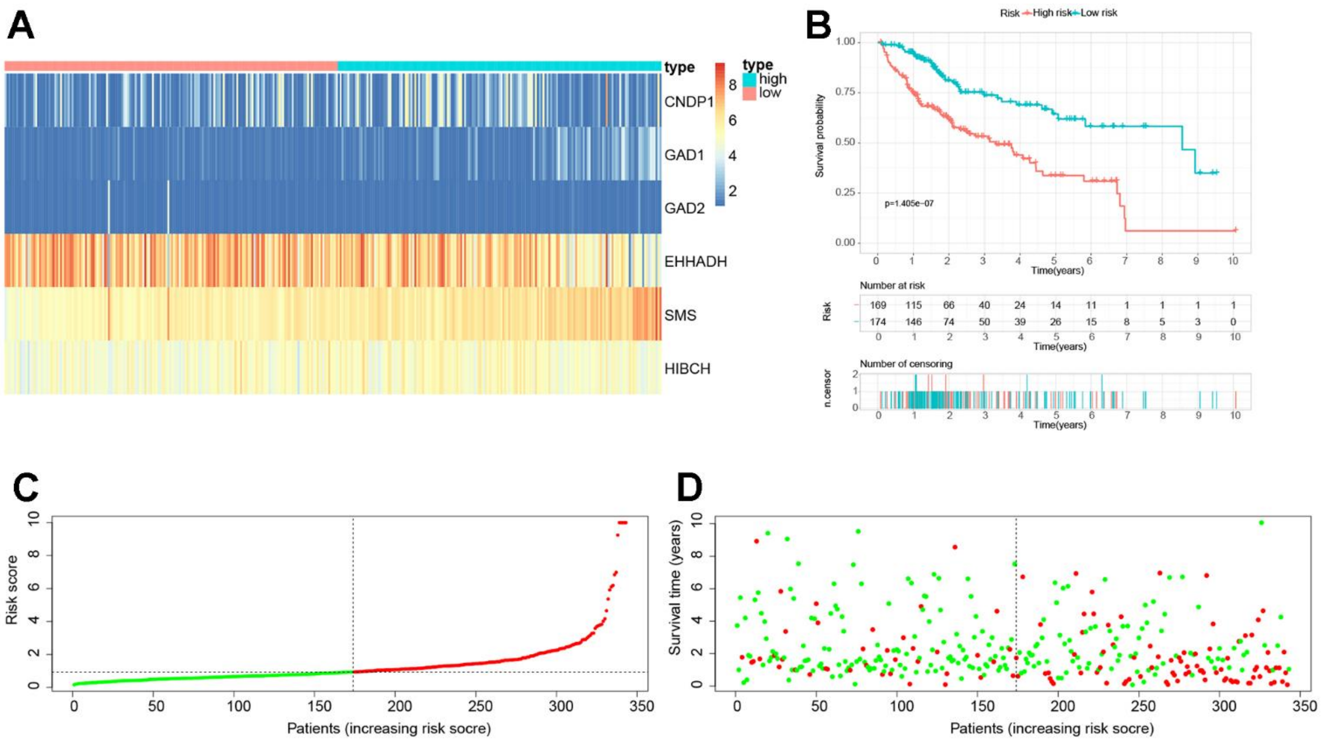

Supplementary Figure 5. Internal validation of the robust βAMRGs-RPS in the test2 cohort. (**A**) Heatmap illustrating the expression levels of six genes between subgroups with high and low risk scores. (**B**) Prognostic prediction depicted by the Kaplan-Meier survival curve comparing subgroups with high and low risk scores. (**C**) Risk score curve plot exhibiting the distribution of individual risk scores, with the patients categorized into low-risk (green) and high-risk (red) groups. (**D**) Risk score scatter plot, where red dots indicate deceased patients and green dots indicate surviving patients.

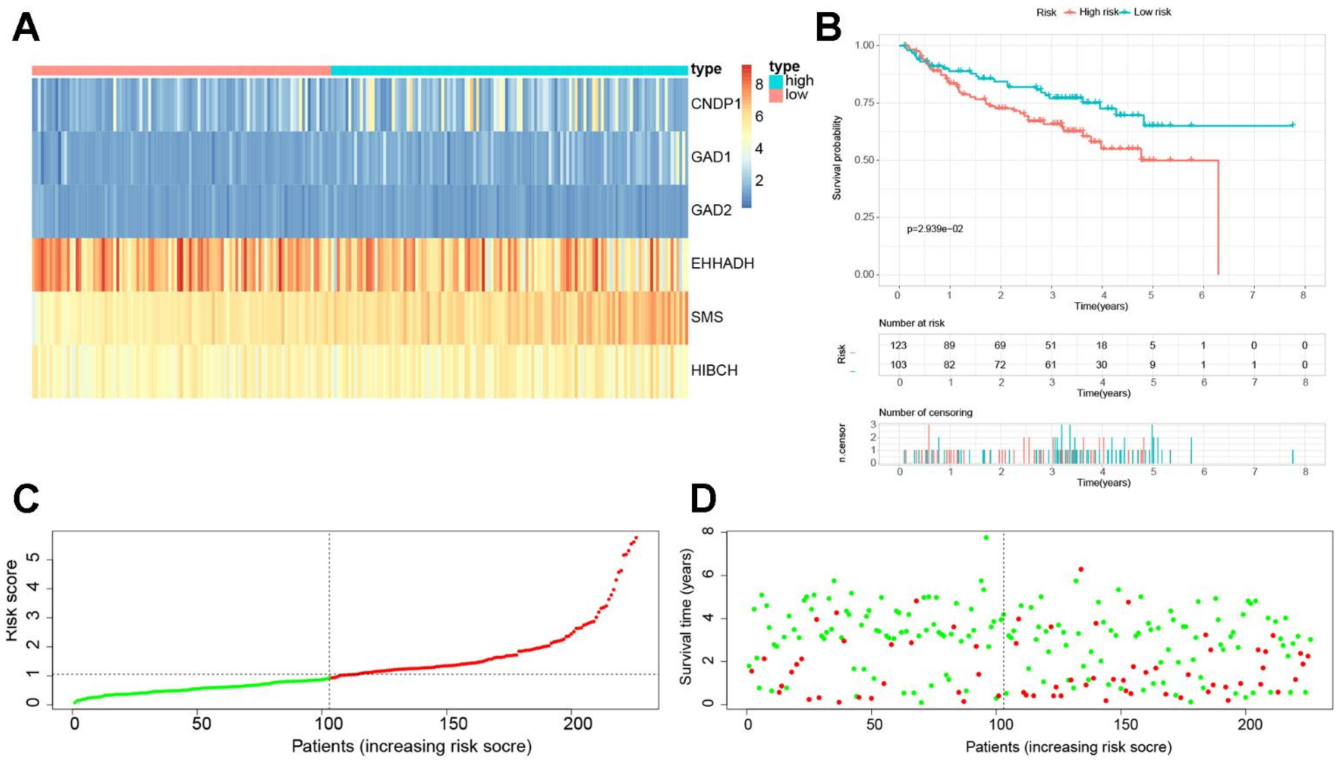

Supplementary Figure 6. External validation of the robust βAMRGs-RPS in the test3 cohort. (**A**) Heatmap illustrating the expression levels of six genes between subgroups with high and low risk scores. (**B**) Prognostic prediction depicted by the Kaplan-Meier survival curve comparing subgroups with high and low risk scores. (**C**) Risk score curve plot exhibiting the distribution of individual risk scores, with the patients categorized into low-risk (green) and high-risk (red) groups. (**D**) Risk score scatter plot, where red dots indicate deceased patients and green dots indicate surviving patients.
